# Systematic parameter estimation in data-rich environments for cell signalling dynamics

**DOI:** 10.1093/bioinformatics/btt083

**Published:** 2013-02-19

**Authors:** Tri Hieu Nim, Le Luo, Marie-Véronique Clément, Jacob K. White, Lisa Tucker-Kellogg

**Affiliations:** ^1^Computational Systems Biology Programme, Singapore-MIT Alliance, Singapore 117576, ^2^Department of Biochemistry, Yong Loo Lin School of Medicine and the National University of Singapore Graduate School of Integrative Sciences and Engineering, National University of Singapore, Singapore 119260, ^3^Department of Electrical Engineering and Computer Science, MIT, Cambridge, MA 02139, USA, ^4^Mechanobiology Institute, National University of Singapore, Singapore 117411 and ^5^Department of Dermatology, State University of New York, Stony Brook, NY 11794, USA

## Abstract

**Motivation**: Computational models of biological signalling networks, based on ordinary differential equations (ODEs), have generated many insights into cellular dynamics, but the model-building process typically requires estimating rate parameters based on experimentally observed concentrations. New proteomic methods can measure concentrations for all molecular species in a pathway; this creates a new opportunity to decompose the optimization of rate parameters.

**Results**: In contrast with conventional parameter estimation methods that minimize the disagreement between simulated and observed concentrations, the SPEDRE method fits spline curves through observed concentration points, estimates derivatives and then matches the derivatives to the production and consumption of each species. This reformulation of the problem permits an extreme decomposition of the high-dimensional optimization into a product of low-dimensional factors, each factor enforcing the equality of one ODE at one time slice. Coarsely discretized solutions to the factors can be computed systematically. Then the discrete solutions are combined using loopy belief propagation, and refined using local optimization. SPEDRE has unique asymptotic behaviour with runtime polynomial in the number of molecules and timepoints, but exponential in the degree of the biochemical network. SPEDRE performance is comparatively evaluated on a novel model of Akt activation dynamics including redox-mediated inactivation of PTEN (phosphatase and tensin homologue).

**Availability and implementation**: Web service, software and supplementary information are available at www.LtkLab.org/SPEDRE

**Supplementary information:** Supplementary data are available at *Bioinformatics* online.

**Contact**: LisaTK@nus.edu.sg

## 1 INTRODUCTION

Dynamic behaviours of biochemical networks can be captured by ordinary differential equation (ODE) models that compute the change of molecular concentrations with respect to time (Fall *et al.*, 2002; Palsson, 2006). For most biochemical pathways with known topology, most reaction rate constants (i.e. the coefficients of the differential equations) are not available from direct experiments. Rate parameters are typically estimated by regression, in other words by fitting the global behaviour of the simulated model to the experimentally observed concentrations. This is a difficult high-dimensional non-linear problem, and search strategies often experience poor convergence and local optima (Kleinstein *et al.*, 2006). The rate parameter estimation problem can naturally be formulated as minimizing a sum of squared errors (SSE), where each error is a difference between simulated concentration and observed concentration, and the summation is over time points and/or experimental treatments. Optimizing this type of SSE objective function can be attacked using a variety of ‘traditional’ global and local search methods: LM (Levenberg–Marquardt, local), SD (steepest descent, local), SRES (stochastic ranking evolution strategy, global), PSO (particle swarm optimization, global) and GA (genetic algorithm, global) (Fogel *et al.*, 1991; Kennedy and Eberhart, 1942; Levenberg, 1944; Marquardt, 1963; Michalewicz, 1994; Runarsson and Yao, 2000). Local and global search methods both have drawbacks, and global–local hybrid searches have also become popular (Ashyraliyev *et al.*, 2008; Fomekong-Nanfack *et al.*, 2007; Rodriguez-Fernandez *et al.*, 2006).

Traditional search methods generate a full vector of rate parameters, simulate the model with this full set of parameters and then accept, reject or adjust the parameters based on how well the simulation agrees with experimental measurements. For networks with few unknown parameters, these ‘simulate-and-match’ methods have been successful at finding good values, or multiple good candidates. The search space for parameter vectors is exponential, and the inevitable trend with any type of exponential growth is that there will eventually be a large enough number of unknown parameters, such that reasonable sampling will not explore very many of the ‘basins of convergence’, and the results will deteriorate. Indeed many high-impact models of biological pathways continue to be built without automating the parameter estimation process (Albeck *et al.*, 2008; Basak *et al.*, 2007; Purvis *et al.*, 2008).

In contrast to standard ‘simulate-and-match’ methods of parameter estimation, spline-based collocation methods have recently been developed that use experimental observations of a protein over time to interpolate the time derivative of the concentration, rather than computing the derivatives based on simulating the ODEs. Traditional methods minimize the violation of experimental observations, subject to obeying the ODE trajectories, while the spline-based collocation methods can be seen as optimizing a dual-like objective function because they minimize the violation of the ODE trajectories, subject to obeying the experimental observations. Note that the spline-based collocation methods require an extensive input dataset with observations for many or all of the proteins. In the past, few large networks had such comprehensive measurements available, but recent trends in proteomic technology (Zhang and Neubert, 2009) suggest that data-rich cases may be increasingly common in the future.

Several spline-based collocation methods have been published recently for the context of biological networks. A spline-based collocation scheme for parameter fitting problems using a modified data-smoothing method and a generalization of profiled estimation was proposed by Ramsay *et al.* (2007). A similar method for problems with high noise and short time-course was introduced by Brewer *et al.* (2008). Zhan and Yeung (2011) used non-linear programming (NLP) to optimize the dual objective. Several ‘decoupling’ strategies (Jia *et al.*, 2011; Vilela *et al.*, 2009; Voit and Almeida, 2004) also use some forms of ‘slope approximation’ from time-series data to avoid doing multiple simulations. Estimating ODE parameters has been studied in the mathematical literature for decades, and an important class of data-rich methods called ‘multiple shooting’ (Bock, 1983; Bock and Schlöder, 1986a, b) has recently been applied to biological networks (Peifer and Timmer, 2007).

To the best of our knowledge, no data-rich parameter estimation methods have implementations publicly available for practical problems with biological networks. The asymptotic runtime of data-rich methods has also been neglected. Many data-rich methods have been published with claims of good accuracy, but to the best of our knowledge, efficiency and runtime have not yet been compared with state-of-the-art, ‘simulate-and-match’ parameter estimation methods.

Scalability with network size is a major remaining challenge in the parameter estimation field, regardless of the objective function or optimization approach. A common strategy for large systems is to decompose the problem. However, the objective functions of parameter estimation are not generally decomposable. Some decomposition approaches exploit specific situations, such as having derivatives available at all timepoints (Chou *et al.*, 2006), or having small sub-networks connected by species with observed concentrations (Koh *et al.*, 2006). The dual-like objective functions of spline-based collocation methods are not readily decomposable, but they do exhibit the important property of sparse interdependence (‘locality’) between the variables. This locality can be a basis for conditional decomposition.

Belief propagation [see (Meltzer *et al.*, 2009) review and (Pearl, 1988) textbook] is an inference method for probabilistic graphical networks with sparse interdependence or locality. It can compute the maximum *a posteriori* (MAP) values for variable parameters in a factor graph, given joint probability distributions that describe the dependencies between adjacent variables. For acyclic graphs, belief propagation guarantees exact optima, and for general graphs, a variant called ‘loopy belief propagation’ (LBP) has had empirical success at approximating the MAP (McEliece *et al.*, 1998; Murphy *et al.*, 1999).

Our method of Systematic Parameter Estimation in Data-Rich Environments (SPEDRE) optimizes the dual objective approximately, via LBP. The innovation is conditional decomposition of the problem into local terms, with pre-computed look-up tables for the discretized solutions to the local terms of the dual objective function. SPEDRE provides dramatic improvement in empirical efficiency, and in effect brings the spline-based collocation (dual objective) methods to the same level of efficiency as the state-of-the-art (primal objective) methods. Asymptotic runtime is polynomial with respect to the number of species, parameters and timepoints in the biological networks, while it is exponential only in the degree of the network.

Finally, we compare the scalability and robustness of SPEDRE against state-of-the-art standalone and hybrid parameter estimation methods, using both a spectrum of artificial cases, and also a novel model of Akt activation based on our previous experimental studies of Akt (Lim and Clement, 2007). Aberrant hyper-activation of the Akt pathway has been detected in up to 50% of all human tumours, and the Akt pathway is an attractive target for anti-cancer drug discovery (Mitsiades *et al.*, 2004). Our model of Akt includes oxidative inactivation of the lipid phosphatase and tensin homologue on chromosome 10 (PTEN), as well as the phosphatidylinositol 3-kinases (PI3K) activation, as competing regulators of Akt in serum-stimulated fibroblasts (Kwon *et al.*, 2004; Testa and Bellacosa, 2001). A more detailed understanding of PTEN dynamics is important because many cancers activate Akt through disruptions of PTEN.

## 2 PRELIMINARIES

### 2.1 Ordinary differential equations

The production and consumption of each species in a biochemical system can thus be described using ODEs. For example, consider a two-species artificial pathway *A⇄B.* Using *k*_1_ (and *k*_2_) to denote the forward (and reverse) reaction rates, we can model the system as follows:
(1)
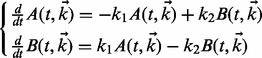



For the simplicity of later figures, our example uses mass action, but more general equations are also permitted. Solving these equations (1) provides the time evolution of the species, dependent on rate parameter vector 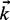. The inverse problem, estimating the rate parameters from observed levels of the species, is a deceptively difficult non-linear optimization problem (Kleinstein *et al.*, 2006). We define the *degree* of each ODE to be the number of terms in its right hand side, analogous to the node degree in a biochemical network diagram.

### 2.2 Rate constant estimation objective

To estimate the rate constants, the most standard (‘primal’) approach is to use a non-linear least squares technique to minimize the weighted SSE objective function
(2)
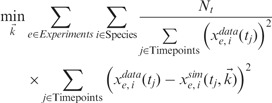

where *N_t_* is the number of observed timepoints per experiment, 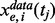 is the observed concentration of species *i* in experiment *e* and 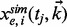 is the simulated level of species *i* in experiment *e* as a function of time and rate constants 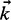. SSE is the most widely used objective for evaluating the success of parameter estimation, but SSE grows with the size of the network. Therefore, we also use the species maximum relative error (MRE), and parameter percentage error (PPE), discussed in Supplementary Text S1 and defined as follows:
(3)
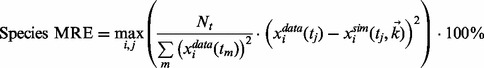

(4)
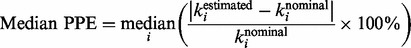

(5)
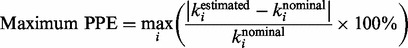



Note that the PPE metric requires the knowledge of the nominal parameter values, which is only feasible in simulated tests.

## 3 ALTERNATIVE OBJECTIVE FUNCTION

### 3.1 Error terms of dual objective function

The error terms of our dual objective function, *ε_e,i,j_*, minimize the disagreement between the right hand sides of the ODEs [which we denote 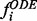, see Equation (1)] and the species derivatives (computed using the species derivatives interpolated from the observed data):
(6)
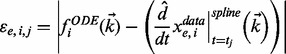

indexed over experiments *e*, species *i* and timepoints *t_j_*. 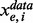 are the time-series observations of species *i* in experiment *e*. The interpolated derivative 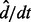 with splines is explained in Supplementary Text S2. The *ε_e,i,j_* terms are similar to the terms of other spline-based collocation methods, but we remark that each *ε_e,i,j_* is defined in terms of a small subset of the parameters 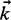, while each term of a ‘primal’ SSE objective uses all parameters via simulation.

### 3.2 Product of functions

Among different schemes for combining error functions into an objective (e.g. sum of squares), we chose multiplication.
(7)
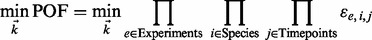



The product of functions (POF) objective will need to be evaluated in practical tests because it has numerical vulnerabilities, such as going to zero if any single term goes to zero. The benefit of using the POF objective is that it is a decomposable expression to facilitate belief propagation for probabilistic inference on a factor graph (Section 4.1). By inspection, the POF is minimized when individual error terms *ε_e,i,j_* are minimized. Assuming the network is low-degree, each *ε_e,i,j_* is a low-dimensional term involving a small subset of the rate parameters 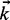. The *ε_e,i,j_* error terms cannot be optimized as independent problems because the subsets of 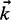 are not mutually exclusive. However, the low dimensionality of *ε_e,i,j_* means we can pre-compute solutions for each *ε_e,i,j_* sub-problem *systematically*. We pre-compute a complete look-up table *T_e,i,j_* that gives the value of *ε_e,i,j_* for each possible (discretized) combination of the relevant 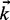 parameters. To combine many low-dimensional systematic *ε_e,i,j_* solutions into an optimal high-dimensional parameter vector is a problem that resembles belief propagation, except with a sparsely connected, cyclic graph instead of an acyclic graph. We next describe in Section 4 how to compute a global estimate of 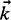 as a graphical inference problem using LBP.

## 4 LOOPY BELIEF PROPAGATION

We now express the POF optimization as an inference problem on a factor graph, in preparation for using LBP to compute the MAP values of the variable parameters, corresponding to the values that minimize the POF objective (Pearl, 1988; Yedidia *et al.*, 2003).

### 4.1 Factor graph

A factor graph is a bipartite graph with factor nodes *ε_e,i,j_* (for each experiment *e*, ODE *i* and timepoint *t_j_*) and variable parameter nodes *k_i_* (for the members of 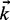). We connect a factor node *ε_e,i,j_* to a parameter node *k_i_* if and only if *k_i_* appears in the equation for *ε_e,i,j_*.

**Fig. 1.**
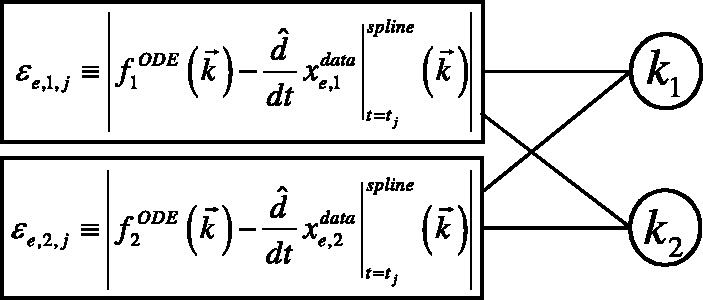
A partial factor graph of the example two-species system, shown for one timepoint *t_j_* and one experiment *e*. The complete factor graph would include factor nodes for every timepoint and every experimental treatment, each connected to the *k*_1_ and *k*_2_ variable nodes

For each factor node, a factor graph has a joint table (or joint probability distribution) to describe the probability of each combination of values for the adjacent variable nodes. We compute a constant look-up table *T_e__,i,__j_*, based on the error term *ε_e,i,j_*, for the joint probability of each combination of the adjacent variable parameters. (Section 4.2 describes how errors are converted into probabilities.) Note that *ε_e,i,j_* represents the discrepancy between the right-hand side of the *i*-th ODE (computed using the variable parameters) and the left-hand side of the ODE (computed from spline-based interpolation of the data for experiment *e*, species *i* and timepoint *t_j_*). Each factor node thus serves to enforce the equality of one ODE at one timeslice and one experiment. An example factor graph corresponding to the two-species *A⇄B* system is shown in Figure 1, with variable nodes (rate parameters) represented by circles, and factor nodes (*ε_e,i,j_*) by rectangles.

### 4.2 Discretization and joint probability tables

Each variable node *k_i_* is associated with a one-dimensional discrete probability distribution, which represents the current belief about the likeliness of that value being in the optimal parameter vector. In theory, variable parameters are real numbers, but to permit us to evaluate combinations ‘systematically’, we discretize the domain of each parameter into a finite number of sub-intervals, or bins, and we represent each bin by its midpoint. Coarser intervals (larger bins) will yield faster runtime at the expense of accuracy. At the start of the algorithm, the one-dimensional discrete probability distribution for each variable parameter is uniform, representing lack of prior knowledge about the value of that parameter. During the process of belief propagation (Section 4.3), the discrete probability distributions will be updated by a message passing algorithm, until convergence or until an iteration limit.

**Fig. 2.**
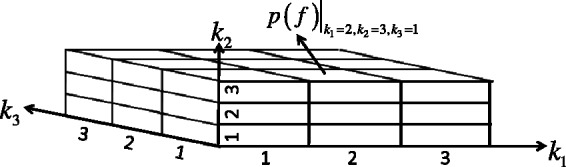
Illustration of a joint probability table of three dimensions, corresponding to rate constants *k*_1_, *k*_2_ and *k*_3_. Each dimension of the look-up table corresponds to one associated variable parameter (variable node in the factor graph), binned into three possible values. The joint probability computed for each cell of the table (e.g. 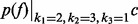 for the cell with *k*_1_ = 2nd bin, *k*_2_ = 3rd bin, *k*_3_ = 1st bin) is obtained by converting the *ε_e,i,j_* error term into a probability, via exponential weighting and normalization

If factor node *ε_e,i,j_* has degree *d*, then the joint table *T_e,i,j_* is a *d*-dimensional table, and each dimension is discretized with the same binning as the variable parameters of the *d* adjacent variable nodes (Fig. 2). To convert the error terms *ε_e,i,j_* into probabilities, we use a Boltzmann-like exponential weighting: 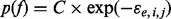. This empirical choice makes large violations exponentially unlikely, and *C* is a normalization constant to make *p*(*f*) a valid probability distribution, i.e. all entries in the discrete joint table sum up to 1. (Alternative methods of representing a systematic solution of *ε_e,i,j_* would also have been possible.)

Note that minimizing the error means maximizing the probability. These probability distributions do not change during the course of belief propagation and can be pre-computed. The error term *ε_e,i,j_* must be computed for every possible discrete combination of the relevant parameters, using the midpoint of each parameter bin, and the errors can then be converted to probabilities to fill in the joint probability table *T_e,i,j_*. The optimal combination of parameters for each *ε_e,i,j_* sub-problem can be found trivially by scanning the *T_e,i,j_* table.

Owing to the discretization required by this method, the estimated value of each parameter is a range rather than a single value. Although discretization sacrifices some accuracy (analogous to round-off error), we choose the variable parameters to be discretized, sometimes quite coarsely, because the output of such a method might be ideal input for a local search method, such as Levenberg–Marquardt or Steepest Descent, to refine afterwards, using a more precise simulation-based objective function.

### 4.3 Loopy belief propagation

Belief propagation can be described informally as two types of message passing: variable nodes *K* pass messages (one-dimensional probability distributions, 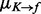) to adjacent factor nodes *f* to communicate what the variable node believes to be the value of its variable. Factor nodes *f* in turn pass a message (one-dimensional probability distribution, 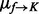) to adjacent variable nodes *K* communicating what they believe the variable values to be. Each message from a factor causes the variable nodes to update their probability distributions, and that update also alters the later messages sent out by the variable nodes. In acyclic graphs, the message passing algorithm yields a provably exact, optimal MAP solution for the variables, efficiently. Murphy *et al.* (1999) describe the LBP algorithm, extending the message passing framework to achieve good heuristic approximations for cyclic graphs. We use a variant of the LBP message-passing algorithm detailed in Koh *et al.* (2007), summarized in Box1 as ‘SPEDRE-base’.

**Box. 1. SPEDRE-base algorithm for computing the MAP estimates on a factor graph. *N*(*node*) is the set of neighbours adjacent to *node*. Steps B.1.1.2 and B.2.1 include a logarithm operation as a heuristic to avoid rounding off small numbers to zero. Because normalizing the messages or probability distributions does not affect the final MAP results (Yedidia *et al.*, 2003), we also perform normalization after the computation of step B.1.1.2 and B.2.1 so that the messages and beliefs are always valid probability distributions at every iteration. The messages history serves as a buffer for incoming messages, and the algorithm makes implicit use of the message history during steps B.1.1.2 and B.2.1.**

**A. Initialization:**

 A.1. Compute look-up joint tables, 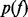, for each factor node

 A.2. Set all variable nodes to uniform distribution

**B. Propagation**: repeat until convergence

 B.1. For each factor node 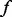

  B.1.1. For each variable node 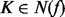

   B.1.1.1. Collect 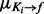: the message from variable node 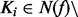 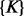 to 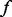, which is 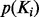, the current probability distribution of 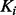

   B.1.1.2. Compute 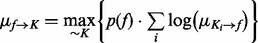

   B.1.1.3. Send 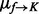 to the message history of 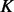

 B.2. For each variable node 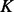

  B.2.1. Update the distribution of 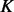 to 
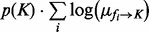
, where 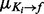 are stored in the message history of 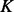

**C. Output**: compute the MAP probability of each variable node

   
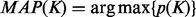

In a discrete joint distribution *g* with dimensions *K*_1_,*K*_2_,..,*K_m_*, we define the maximization over a dimension *K_i_* as follows:
(8)
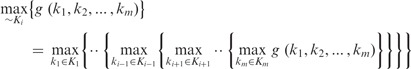

where ∼*K_i_* denotes the set of all dimensions in *g* except *K_i_*.

Convergence in step B occurs when no normalized message in the current iteration differs by more than a tolerance value from the corresponding message in the previous iteration. As the LBP algorithm is not guaranteed to converge, and in some cases might oscillate (Murphy *et al.*, 1999), we impose an additional criterion to limit the total number of iterations.

From Box 1, the asymptotic runtime of the modified LBP algorithm, including pre-computation of all the look-up joint tables, can be expressed as:



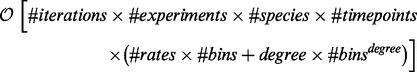



See Supplementary Text S3 for a derivation of the asymptotic runtime. The time complexity scales exponentially with the factor graph degree, which is defined as the maximum number of variable nodes adjacent to any factor node, or the maximum number of unknown rate parameters appearing in any ODE. For a factor graph with bounded degree, the method scales polynomially with respect to the number of species, timepoints and discrete bins. This means the method scales well on biological pathways with a small bounded number of reactions per species. Our asymptotic runtime compares favourably with conventional (‘primal’) methods, because primal methods search for full-length parameter vectors in a space that grows exponentially with the number of parameters. Although primal methods with heuristic sampling do not have to cover the entire parameter space, they must maintain some coverage of the major ‘valleys’ of the objective function. If the number of valleys and inflection points grows with the size of the parameter space, then primal methods will perform poorly (accuracy versus runtime) on large networks.

## 5 Results

**Fig. 3.**
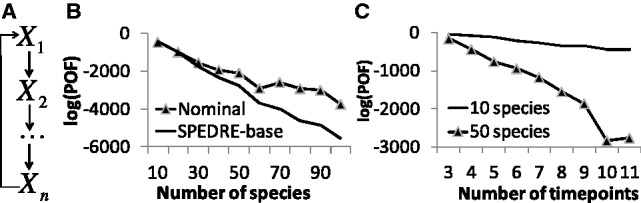
(**A**) Ring network diagram; (**B**) normalized log(POF) with respect to the number of timepoints on different network sizes; (**C**) normalized log(POF) of LBP-predicted rate constants, versus the normalized log(POF) of nominal (‘correct’) rate constants on circular networks of 10 to 100 species with 11 timepoints. (Total simulation duration was 4 s, with 0.4 s for each time step). Each rate constant was discretized into 10 equidistant bins from 0.05 to 1.05, with the nominal rate constants selected from among the bin midpoints. Dataset appears in Supplementary Source Files

Before testing the overall performance of the method on full problems, we first performed simplistic tests with partial problems, to isolate specific variables of interest such as #*species* and #*timepoints*. We monitored performance as a function of network size and timepoint spacing, to probe two sources of potential error in the SPEDRE-base method: spline accuracy and the POF objective function. The simplistic tests used ring-shaped networks (Fig. 3A), with nominal parameters randomly chosen to be at exact mid-points of the parameter discretization bins. Simulated data were generated with random initial concentrations, using the nominal rate parameters. For each run of SPEDRE-base, we monitored the objective
(9)
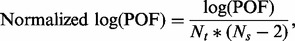

normalized with respect to the number of factors in the product of Equation (9). The objective declined when the number of timepoints increased (Fig. 3B), indicating as expected that SPEDRE gives better estimates of the parameters when timepoints are densely sampled.

Next we compared the normalized log(POF) value between the results of SPEDRE-base, and the nominal (‘correct’) rate constants, using 10 time steps (i.e. 11 timepoints) for each test. Figure 3C shows that the POF scores of the exact nominal parameters were higher than the POF scores of the parameters found by SPEDRE-base, for all networks of significant size. Because SPEDRE-base found parameters with better scores than the ‘correct’ parameters, we infer that the POF objective is an imperfect score of parameter accuracy.

### 5.1 Scalability with artificial networks

The complete SPEDRE method consists of SPEDRE-base (Sections 3–4) followed by LM to refine the discretized results from SPEDRE-base. The major task of SPEDRE-base is to find good starting values for local optimization, and post-fitting is a necessary part of the procedure. We compared the performance of the complete SPEDRE method against a selection of popular local, global and hybrid methods, on parameter estimation problems from low-degree networks. All tests were full problems with parameters set randomly, without regard to bins or bin midpoints. Random networks of increasing size (from 30 to 150 species) were constructed with low degree (two-thirds of the reactions involving three species, and one-third involving two species), to generate parameter estimation problems with increasing scale. We tested 14 parameter estimation methods including SPEDRE-base, LM (local), SD (local), SRES (global), PSO (global) and GA (global), plus hybrid global–local combinations of these methods (e.g. SRES_LM denotes the global method SRES plus the local method LM). Runtime and multiple error scores were measured as a large-scale screen (see Supplementary Text S5, Supplementary Figs S6 and S8 and Supplementary Tables S7 and S9). The best-performing methods (asterisks in Supplementary Fig. S6) are consistently SPEDRE and a subset of hybrid stochastic-local methods. Highly similar scores can occur when multiple methods converge to the same local optimum. Accuracy tests showed the following trends: (i) for small networks, local methods performed well; (ii) hybrid methods (including SPEDRE) showed superior accuracy to standalone local or global methods; (iii) SPEDRE accuracy was comparable with the accuracy of other hybrid methods; (iv) the quality scores of SPEDRE-base were significantly worse than SPEDRE, confirming that the LM post-processor is indeed important for refining discretized results. Supplementary Text S5 also clarifies the trade-off between accuracy and speed. We conclude that SPEDRE was ‘tied’ with other state-of-the-art methods, for the spectrum of low-degree data-rich problems we constructed.

### 5.2 Performance on a novel model of Akt pathway activation

**Fig. 4.**
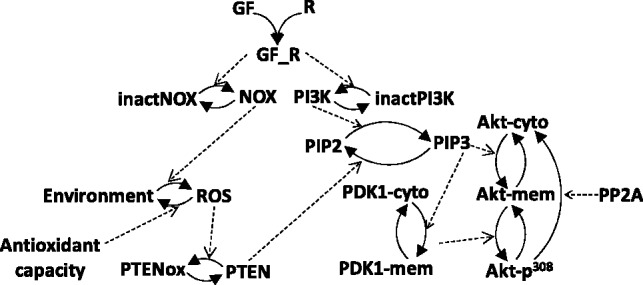
Network diagram of the Akt model, including redox regulation of PTEN. The prefix ‘inact’ denotes inactive species; the suffix ‘cyto’ (or ‘mem’) indicates cytosolic (or plasma membrane) localization. The suffix ‘p^308’^ indicates phosphorylation at residue Thr^308^

To test SPEDRE on a realistic biological pathway, we built a novel model of Akt activation (‘Akt model’), using our experimental data from recent studies (Lim and Clément, 2007). Akt is a kinase, frequently over-activated in cancers, that signals for survival and proliferation. The signalling events in our model of Akt activation are illustrated in Figure 4 and described below. Akt is activated after stimulation by serum (growth factors), not only via the canonical activation of PI3Kinase (PI3K) by serum, but also via reactive oxygen species (ROS) and ROS-induced inactivation of the phosphatase PTEN. ROS are produced by NADPH oxidase (NOX), and degraded by anti-oxidants. The phosphorylation and activation of Akt is a multi-step process, involving the translocation of Akt from the cytosol to the cell membrane and its phosphorylation by the kinase PDK1 at Thr^308^. The translocation of Akt and PDK1 to the membrane is controlled by the level of phosphatidylinositol 3,4,5-trisphosphate (PIP3), which is determined by the balance between PIP3 production (by serum-activated PI3Kinase) and PIP3 degradation (by the phosphatase PTEN). Phosphorylated Akt returns to the cytosol and is subject to dephosphorylation by PP2A.

The model was built manually based on dynamic measurements of the Akt pathway, observed in serum-starved mouse embryonic fibroblasts stimulated by the addition of 10% serum to the culture medium (Lim and Clément, 2007). Supplementary Table S11 defines the full model.

**Fig. 5.**
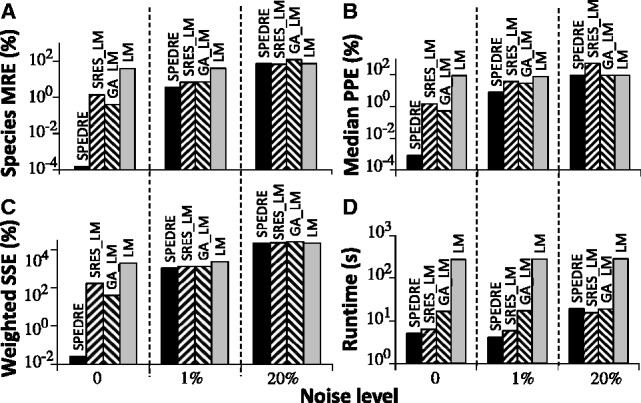
Comparison of parameter estimation algorithms applied to the Akt network, with noise levels 0, 1 and 20%, evaluated by (**A**) species MRE, (**B**) median PPE, (**C**) weighted SSE and (**D**) runtime

Using simulation, we generated complete datasets with artificial noise at levels of 0, 1 and 20%. Parameter estimation performance was compared between SPEDRE and some popular methods (Fig. 5, with complete results in Supplementary Table S12).

According to Figure 5A–C, the prediction quality of SPEDRE (leftmost bar, shaded black) is better than the other methods for noise-free and 1%-noise datasets. With noise levels of 20%, all methods perform unacceptably, providing worse than 100% species MRE, as shown in Figure 5A. Similarly, on the 20%-noise data set, all methods have unacceptably high median PPE and weighted SSE, as shown in Figure 5B–C. In this parameter optimization test with Akt dynamics, SPEDRE out-performed other methods, indicating that the parameter neighbourhoods it gave to LM were better than the neighbourhoods from other global methods, even though SPEDRE-base had equivalent performance in non-biological tests.

Figure 5D also displays a counterintuitive phenomenon, in which the local search method LM takes longer to run than any hybrid method including LM as a post-processor. LM performs many iterations if it starts with a random guess, but it converges quickly if it starts with the output of a global search method. Adding more phases of search would be expected to increase rather than decrease the total runtime, but in this case, a phase of global search led to faster LM convergence, which more than compensated for the time of running the global search.

## 6 DISCUSSION

The key innovations of SPEDRE are the use of a probabilistic graphical model to decompose the dual objective function, and pre-computation of discrete solutions to each sub-problem. The method has a well-defined asymptotic runtime and good scalability, in exchange for approximate heuristic optimization.

The SPEDRE approach aims for asymptotic scalability at the expense of accuracy. This philosophy appears in (i) the use of splines to approximate the species derivative, (ii) the use of binning to discretize the parameter space and (iii) the use of LBP for probabilistic inference. Each of these elements can introduce error. We believed the dangers of compounded errors would make the SPEDRE method less robust to noisy data than simulation-based methods. The expected sensitivity of SPEDRE to input noise has not yet been confirmed in the tests shown (and in other tests, such as Supplementary Table S12); rather we found that all methods gave unacceptably poor answers with noisy data. Future work must continue to characterize the numerical stability, approximation error and noise tolerance of SPEDRE and other parameter estimation methods. SPEDRE is in fact a general algorithm for ODE parameter fitting, applicable to any case in which the right-hand sides of the ODEs have few terms (low degree), and where the data-fitting problem provides dense observations of all variables.

The accuracy and speed of SPEDRE were compared against several methods of parameter estimation, in low-degree, data-rich test cases. SPEDRE performance was competitive in all tests, and SPEDRE was the best-performing method for the Akt network test. We conclude that SPEDRE performs well when tested in the specific niche of problems for which it was designed. Naturally the SPEDRE performance would degrade (perhaps exponentially) outside of its intended niche. SPEDRE exhibits an abrupt trade-off between problem type and performance, but performance trade-offs are not new to parameter estimation research. Major pathway simulation software packages already maintain collections of multiple parameter estimation methods, rather than expecting a single best method to cover all problems.

A current hurdle for broader applicability of SPEDRE is the inability to handle high-degree nodes. Many small networks are low-degree, but large networks often have at least one hub. In order for new spline-based collocation methods to be truly superior to conventional (‘primal’ objective) parameter estimation methods, they would have to handle high-degree networks and extensive gaps in experimental observations, robustly. Future innovations may be able to develop a new composition of parameter estimation methods, so that low-degree sub-problems can be solved by SPEDRE and high-degree hubs can be treated separately.

A side-effect of our work is to provide performance comparisons for several hybrid and standalone parameter estimation methods. Our tests reproduced the earlier observation that hybrid methods generally perform better than standalone global methods (Ashyraliyev *et al.*, 2009; Rodriguez-Fernandez *et al.*, 2006). One surprising phenomenon we observed was that performing a global search prior to a local search sometimes caused the total runtime to be faster than using the local search alone (Fig. 5D). Future work may be able to exploit this non-additive runtime effect, perhaps through deeper integration of global and local search methods, rather than applying independent methods sequentially.

A distinguishing feature of SPEDRE is that it requires large amounts of concentration measurement data, which would have been prohibitive a decade ago. Traditional experimental methods required an investment of labour and resources that was roughly linear in the number of proteins studied. New proteomic methods can measure additional proteins at virtually no additional cost, and proteomic datasets are starting to provide data-rich environments with measurements of all proteins in a system. SILAC technology has recently been used for time-series measurements of 147 proteins (Tasaki *et al.*, 2010) in NIH3T3-derived cells, and again for time-series of 534 proteins in the cytosol and 626 proteins in the nucleus in glucocorticoid-exposed myogenic cells (Reeves *et al.*, 2009). Most proteomic studies have not been performed with time-series repeats for studying dynamics, but large-scale dynamic data will become increasingly available with the explosive growth in the number of proteomic experiments (Zhang *et al.*, 2011). New studies of large networks will give rise to huge parameter estimation problems, with rich datasets, but with too many unknown parameters for conventional methods to solve.

We believe that proteomic technology both enables and requires novel approaches to parameter estimation such as SPEDRE. As models grow in size owing to technological advances, decomposition-based methods will probably dominate non–decomposition-based search methods, which suffer from the curse of dimensionality. The trade-offs exhibited by our method may be increasingly desirable for future trends in parameter estimation.

*Funding*: Lee Kuan Yew Postdoctoral Fellowship and grant R-252-000-342-112 (to L.T.-K.); Singapore-MIT Alliance grant C-382-641-004-091 (to L.T.-K. and J.K.W); grant BMRC 09/1/21/19/603 (to M.-V.C.)

*Conflict of Interest*: none declared.

## Supplementary Material

Supplementary Data
