# Supplementary material for: Systematic parameter estimation in data-rich environments for cell signalling dynamics
Source: Bioinformatics. 2013 Feb 19;29(8):1044–51. doi: 10.1093/bioinformatics/btt083 (PMC3624804; doi:10.1093/bioinformatics/btt083)
Supplement: Supplementary Data [file supp_29_8_1044__index.html]

Systematic parameter estimation in data-rich environments for cell signalling dynamics — Systematic parameter estimation in data-rich environments for cell signalling dynamics — Supplementary Data 

# Systematic parameter estimation in data-rich environments for cell signalling dynamics

## Supplementary Data

files

**Files in this Data Supplement:**

- Supplementary Data - pdf file
- Supplementary Data - zip file
