# Supplementary material for: Systematic parameter estimation in data-rich environments for cell signalling dynamics
Source: Bioinformatics. 2013 Feb 19;29(8):1044–51. doi: 10.1093/bioinformatics/btt083 (PMC3624804; doi:10.1093/bioinformatics/btt083)
Supplement: Supplementary Data [file supp_btt083_SPEDRE_Supplement.pdf]

Systematic Parameter Estimation in Data-Rich Environments (SPEDRE) for Cell Signaling Dynamics

Tri Hieu Nim[1], Le Luo[2], Marie-Véronique Clément[2], Jacob K. White[1,3], Lisa Tucker-Kellogg[1,4,5*]

[1]Computational Systems Biology Programme, Singapore-MIT Alliance, Singapore 117576.

[2]Department of Biochemistry, Yong Loo Lin School of Medicine and the National University of Singapore Graduate School of Integrative Sciences and Engineering, National University of Singapore, Singapore 119260

[3]Department of Electrical Engineering and Computer Science, MIT, Cambridge, MA, USA 02139.

[4]Mechanobiology Institute, National University of Singapore, Singapore 117411

[5]Department of Dermatology, State University of New York at Stony Brook, NY, USA 11794.

SUPPLEMENTARY TEXT

S1. Motivations for Choice of Metrics Used in Benchmark Comparison

We assessed the parameter estimation quality using the species maximum relative error (species MRE), a dimensionless metric. Species MRE is an improvement over SSE in this case because MRE does not depend on size of the system, and therefore can evaluate the ability to match data over a range of network sizes. The weight $\frac{N_t}{\sum_m \left(x_{e,i}^{data}(t_m) \right)^2}$, the inverted mean squared of the data for all timepoints used in equation (3), makes the measure equivalently sensitive to relative errors in any species regardless of whether the species has high or low concentration. Species MRE is a preferred choice in this work, as it specifies the maximum distance between the simulated and observed trajectories for all species.

If the SSE objective function has multiple minima, different sets of parameters may match the same set of data equally well. Thus we also used maximum and median PPE (parameter percentage error) as additional metrics to assess a parameter estimation method, evaluating whether the underlying parameters were indeed recovered. This is only feasible for simulated test cases, where the parameter values are known.

S2. Spline Approximation of Derivatives

SPEDRE allows the user to select either cubic clamped spline or smoothing splines for interpolation of data points. The results in the main text (SPEDRE and SPEDRE_base) used clamped cubic splines. Clamped cubic spline interpolation approximates the first derivatives using three data points and two end-point derivatives (Tewarson, 1980). End-point derivatives make the computation of the spline unique. Fig. S2.1 illustrates the derivative for species x_i at timepoint t_j, approximated (dashed arrow) using a clamped cubic spline based on observed data for t_{j-1}, t_j and t_{j+1}; and endpoint derivatives from the ODEs at timepoints t_{j-1} and t_{j+1}. We estimated the endpoint derivatives by ting $f_i^{ODE}\left(\vec{k}\right)\Big|_{t=t_{j-1}}^{e}$ and $f_i^{ODE}\left(\vec{k}\right)\Big|_{t=t_{j+1}}^{e}$, the right hand side of the ODE for species i, experiment e at timepoint t_{j-1} and t_{j+1}, respectively. Other spline methods could also be used to compute the approximate derivatives.

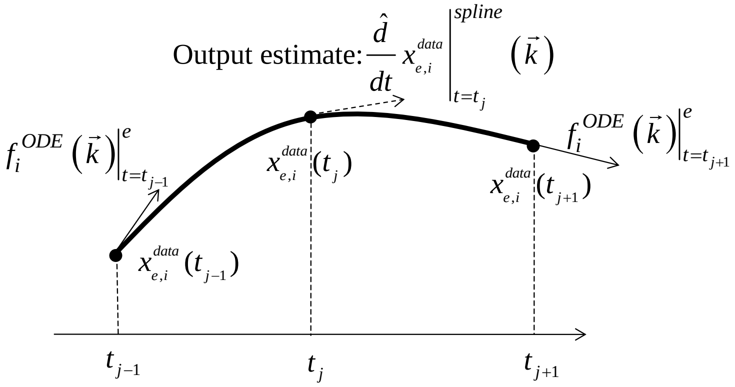

Figure S2.1. Illustration of computing the approximate derivative using clamped cubic spline interpolation

Smoothing splines, following De Boor's approach (De Boor, 1994) were implemented in addition to cubic spline and can be enabled in SPEDRE. Figure S2.2 describes the derivative for species x_i at timepoint t_j, approximated (dashed arrow) using a smoothing spline based on observed data $x_{e,i}^{data}(\vec{t})$, where $\vec{t}$ represents the vector of all timepoints where measurements are available for species x_i.

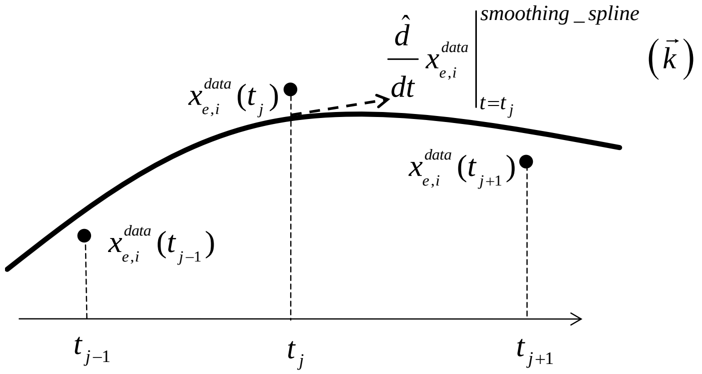

Figure S2.2. Illustration of computing the approximate derivative using smoothing spline approximation

While the cubic spline requires only three neighboring datapoints, the smoothing spline was constructed based on concentration values of each species over all timepoints. A user-defined smoothness factor p (default value 0.95) adjusts the balance between having a smooth curve versus being close to the given data. The performance of SPEDRE with cubic splines (SPEDRE) and with smoothing splines (SPEDRE-S); and the performance of SPEDRE-base with cubic splines (SPEDRE-base) and with smoothing splines (SPEDRE-base-S) are shown in Table S10. During these tests, SPEDRE performance was not improved by the use of smoothing splines, but in general smoothing splines are often better than cubic splines at interpolating from noisy data points. Both spline options have therefore been made available to fit the needs of different users.

S3. Asymptotic Analysis of the Modified LBP (SPEDRE-base) Algorithm

To determine the run time for step A in Box 1, we need to find the size of each lookup table and the number of lookup tables that need to be computed. Each dimension of the joint table corresponds to one variable node, which is one reaction in the corresponding ODE. Hence the number of dimensions of the joint table, which is the degree of the factor graph, is proportional to the number of reactions in the ODE. The size of a joint table is $\mathcal{O}$ (*#bins*^{degree}). The factor graph defines one factor node for each ODE, for every experiment and at every timepoint excluding the two end timepoints. Therefore, the number of joint

tables is the product of the number of ODEs (or species), experiments, and timepoints (=*#species×#experiments×#timepoints)*. Therefore, the required time for step A is:

$$\mathcal{O}(\#species \times \#experiments \times \#timepoints \times degree \times \#bins^{degree})$$

From step B.1 in Box 1, for every iteration, each factor node iterates through each of the neighboring variable nodes and performs multiplication. This is equivalent to the multiplication operation between a joint tables and a one-dimensional discrete distribution, which takes the run time proportional to the size of the joint table, or $\mathcal{O}(\#bins^{degree})$. Since there are *degree* variables connected to the factor node, the computation requires $\mathcal{O}(degree \times \#bins^{degree})$ operations. Thus the time required for step B.1 is $\mathcal{O}(\#species \times \#experiments \times \#timepoints \times degree \times \#bins^{degree})$. From step B.2 in Box 1, for every iteration, each variable node needs to compute its new distribution based on the messages from the factor nodes. As there are *#rates* variable nodes, the maximum the number of computed messages is *#species*×*#experiments*×*#timepoints* and each message has size #bins, so the time required for step B.2 is:

$$\mathcal{O}(\#species \times \#experiments \times \#timepoints \times \#rates \times \#bins)$$

To run on all the factor nodes for #iterations iterations, the time required for step B is:

$$\mathcal{O}(\#iterations \times \#species \times \#experiments \times \#timepoints \times (\#rates \times \#bins + degree \times \#bins^{degree}))$$

Since the run time for step B.1 and B.2 dominates that of step A and step C, the total time complexity of the LBP algorithm (SPEDRE-Base) is:

$$\mathcal{O}(\#iterations \times \#species \times \#experiments \times \#timepoints \times (\#rates \times \#bins + degree \times \#bins^{degree}))$$

The asymptotic analysis reveals an interesting property of the method, in which the time complexity only scales poorly with the factor graph degree. This means with a factor graph with a small bounded degree, the method scales well with respect to the number of species, timepoints and discrete bins. Correspondingly, this means the method scales very well on biological pathways with a small, bounded number of reactions per species.

S4. Benchmark Comparison – Experiment Setup

As described in main text, we propose that the SPEDRE-base algorithm should be followed by a local search method such as LM, because a hybrid approach can correct some of the fine-grained inaccuracies created by the discretization and by the numerically approximate derivatives. SPEDRE-base is written in C++, the same language as the Copasi parameter estimation tools (Hoops, *et al*., 2006). A wide variety of parameter estimation algorithms assert claims of supremacy, and algorithms in the "evolutionary strategies" family are particularly well-reviewed (Moles, *et al*., 2003). For a fair comparison, we chose a variety of standard methods:

- Local search: Steepest Descent (SD), Levenberg-Marquardt (LM)
- Global search: SPEDRE-base, Genetic Algorithm (GA), Evolution Strategy using Stochastic Ranking (SRES), Particle Swarm Optimization (PSO)

Four forms of global and two forms of local optimization produce 8 hybrid methods: SPEDRE, SRES_LM, PSO_LM, GA_LM, SPEDRE-base_SD, SRES_SD, PSO_SD and GA_SD. Note that hybrid methods GA_LM and PSO_LM were previously proposed by Katare *et al*. (Katare, 2004). Rodriguez-Fernandez *et al*. also proposed the hybrid of SRES with a local optimizer (Rodriguez-Fernandez, *et al*., 2006). The implementations of the comparison algorithms were used through the Copasi software package (version 4.4, build 26). Each parameter estimation method was run with custom configuration (*e.g.* number of generations, number of iterations, *etc.*) such that all methods take a similar amount of time to complete. However, this constraint may not always hold (see Table S7) as local search methods and GA can converge very quickly, regardless of user-defined iteration limits. Copasi (.CPS) source files are available as Supplementary Source Files, describing the model parameters and configuration for each parameter estimation method on each test case (including the ring networks, artificial networks and the Akt model). Experiments were performed on an Intel® Core™ i7 2.8GHz CPU and 4GB memory workstation running Windows® XP™.

S5. Benchmark Comparison on a Series of Artificial Networks

We compared the performance of all methods described in Supplementary Text S4 on a series of randomly constructed low-degreed networks, including run time, species maximum relative error (MRE), median and maximum parameter percentage error (PPE). The networks vary in size from 30 species to 150 species. Data sets were obtained by simulating the networks with nominal reaction rates, to which Gaussian noise with standard deviation of 20% times the nominal values or no noise was added to generate simulated observations. A sample network of size 30 is shown in Figure S5.1. Fig. S6 showed the performance of all standalone and hybrid methods on an artificial network with 130 species and 130 reaction rates (each bar showing the average with error bar indicating standard deviation from three tests with different parameter sets using the same network topology), where complete comparison results on different network sizes are shown in Table S7. To score the quality of the parameter estimation results, we used an additional metric called weighted SSE, defined as:

$$\sum_{e\in Experiments}\sum_{i\in \text{Species}}\sqrt{\frac{N_t}{\sum_{j\in \text{Timepoints}}\left(x_{e,i}^{data}(t_j)\right)^2}}\sum_{j\in \text{Timepoints}}\left(x_{e,i}^{data}(t_j)-x_{e,i}^{sim}(t_j,\vec{k})\right)^2,$$

which follows the default SSE objective function used in the Copasi software package. All metrics were normalized such that the highest score for any method is 1.0. Note that low runtime indicates good scalability, and low species MRE, median and maximum PPE indicate good accuracy.

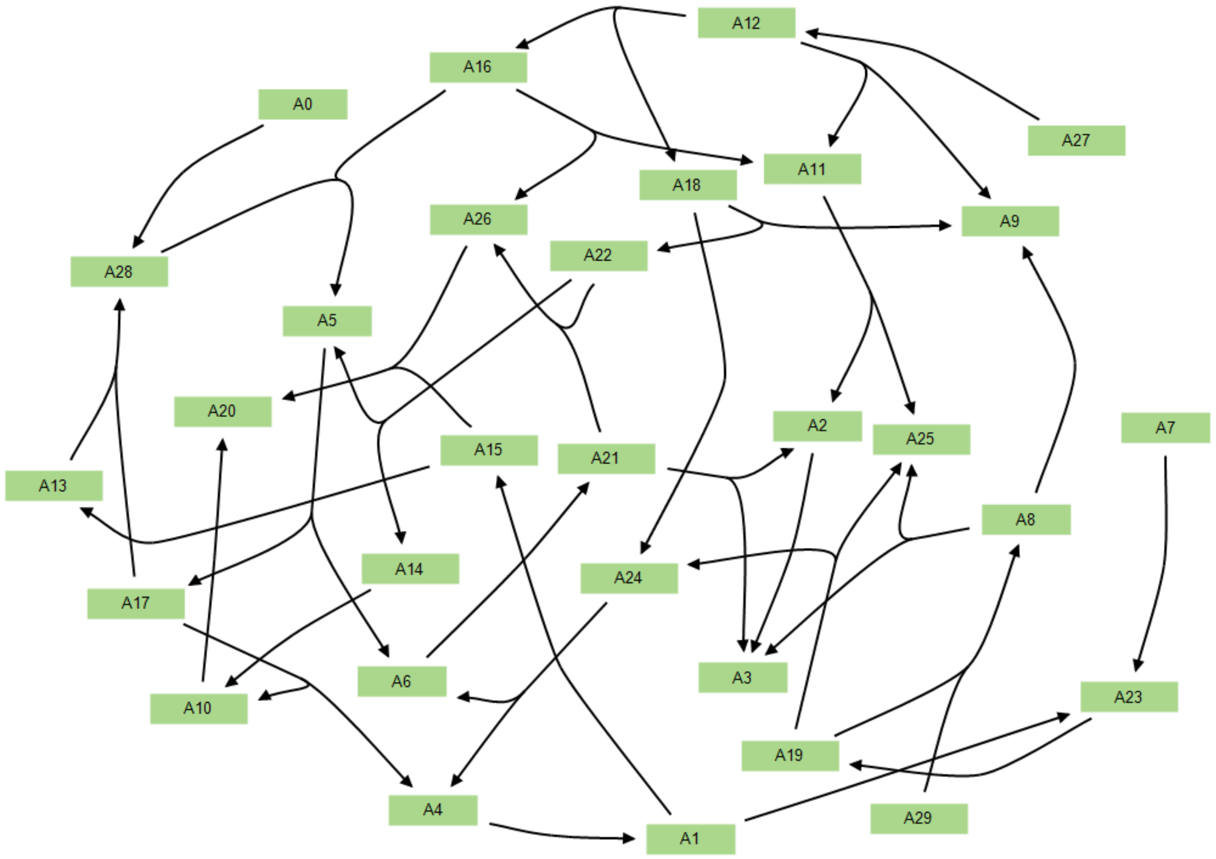

Figure S5.1 – Network diagram for a random artificial low-degreed network of size N=30, with 30 species and 30 reaction rates. Random reactions (with two-thirds of the reactions involving 3 species, and one-third involving 2 species) were added between the species such that the maximum degree for each node is three.

Here we provide further analysis of the performance comparison in artificial networks (Fig. S6 and Table S7) to accompany section 5.1 of the main text. The speed and accuracy of stochastic global methods (e.g., SRES, PSO, GA) depend on the degree of sampling, where greater sampling gives better quality

at the expense of runtime. To compare sampling methods fairly, we chose all user-defined iteration limits to allow the sampling-based (global or hybrid) methods to run for at least the same length of time as SPEDRE (-base or hybrid), prior to comparing accuracy. However it was often not possible to ensure equal runtime. SRES and its hybrids usually required additional runtime on large networks, and PSO sometimes suffered poor scaling as well. The GA method always converged very quickly, regardless of user-defined iteration limits, but this is not necessarily an advantage because the accuracy of GA (and GA hybrids) was significantly worse for large networks. A full assessment of comparative performance therefore requires a holistic view of the trade-off between runtime and accuracy.

Accuracy comparison based on the size-130 error-free test case (Fig. S6A, asterisks) showed SPEDRE to be among the best scoring methods. Tied with SPEDRE were two hybrid global-local methods SRES_LM and PSO_LM. Error bars on these three methods are negligible, indicating that the performance was consistent across the three random replications of the test. Note that SRES_LM, PSO_LM, and SPEDRE, in addition to comparable accuracy, showed comparable runtime (first column group). It should be noted that LM had much worse performance (blue columns marked with LM label) when not seeded with an initial guess from global methods such as SPEDRE-base, SRES or PSO.

For the 20%-noise test case, Fig. S6B showed a set of 5 methods (marked with asterisks), including SPEDRE, that were tied for best performance. This comparative result should be interpreted with awareness that all methods had poor absolute accuracy (see Table S7B, column 130).

For test cases of size below 100 (Table S7A, column 30-90), the local search method LM easily achieved best accuracy, as did any LM-hybrid methods (rows for species MRE, maximum PPE, median PPE and weight SSE, cells highlighted in red). For networks of size 100 and above (columns 100-150), LM performed worse than any LM-hybrid method, with the exception of the size-110 test case. In test cases of size 110 to 140 (column 110-140), SPEDRE was tied with PSO_LM in accuracy (species MRE row, cells highlighted in red) and both were not worse than any other method. Test cases of size 100 and 150 were the only large cases where SPEDRE did not achieve the best score.

As expected, introduction of 20% noise (Table S7B), caused worse accuracy on most methods, compared with noiseless tests (Table S7A). For tests with 20% noise, SPEDRE and SRES_LM were the best performing methods for large test cases, according to the species MRE score (Supplementary Text S1), except in the test of size 140 (Table S7B, comparing the Species MRE set of rows, with best scores in red). In the size-140 test case, SPEDRE scored worse than other LM-hybrid methods, but SPEDRE-base_SD achieved the same best score as other LM-hybrid methods (column 140, species MRE row, cells highlighted in red). Based on other measures of accuracy (maximum PPE, median PPE and weighted SSE), the best scoring method may vary between test cases (maximum PPE, median PPE and weighted SSE, cells highlighted in red), but most best-scoring methods are LM-hybrid methods. We thus conclude that SPEDRE was "tied" with other state-of-the-art methods, for the spectrum of low-degree data-rich problems we constructed.

A separate series of tests were performed to assess the performance of all methods with network sizes from 120 species to 150 species (Table S8 and Figure S9). Three different network structures were constructed for each network size, and simulated data were generated at different noise levels (5%, 10%, and 20%). From Figure S9, we observed that LM had similar performance as any LM-hybrid methods, all achieving the best score based on weighted SSE and species MRE metrics. However, from earlier tests using noiseless datasets (Table S7A), the LM method was shown to break down for certain tests while LM-hybrid methods were more robust. As the runtime of all methods were kept to be of the same order of magnitude, the accuracy comparison shows that SPEDRE was tied to all other LM-hybrid parameter estimation methods.

SUPPLEMENTARY FIGURES AND TABLES

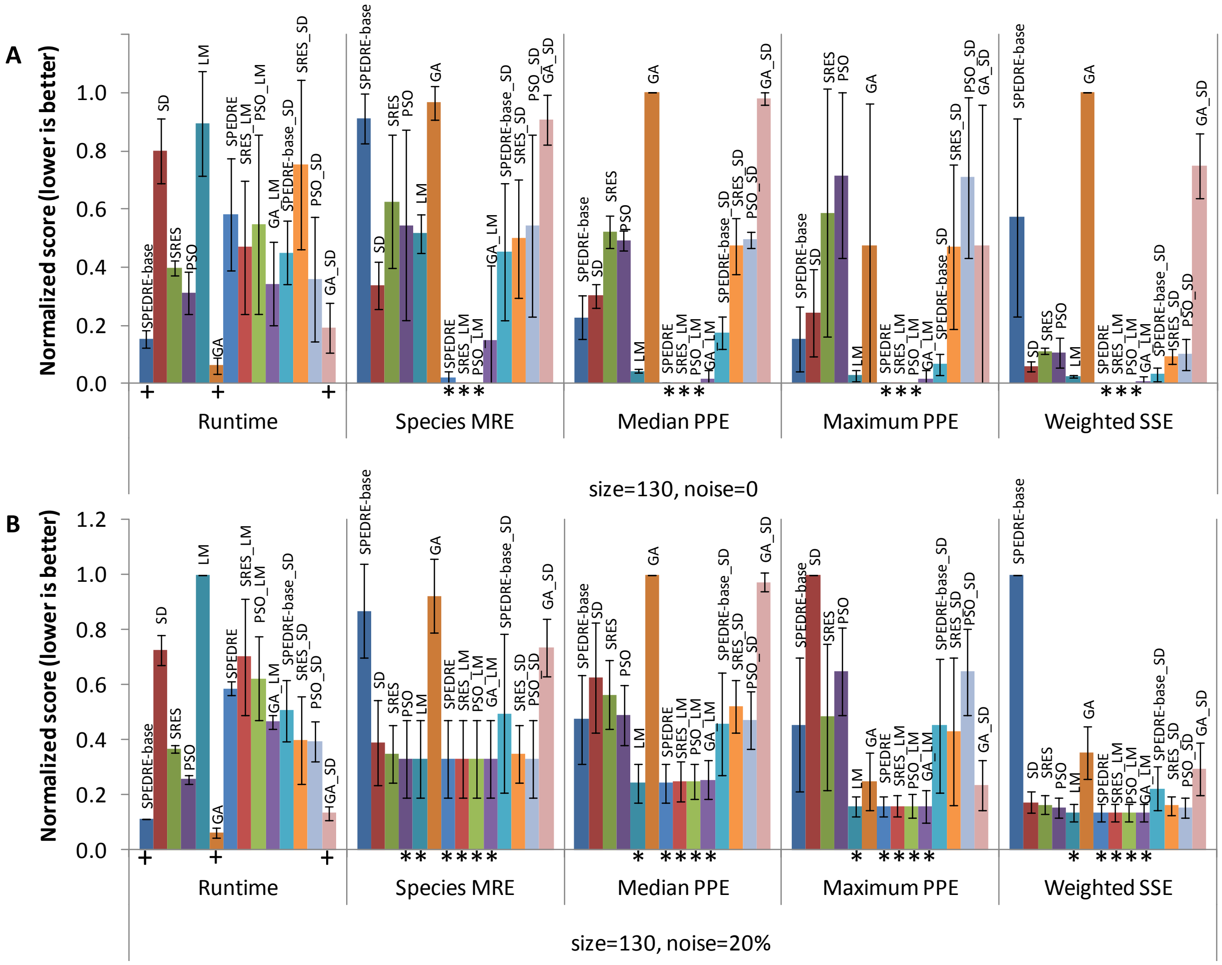

Figure S6 - Comparison of standalone and hybrid methods on artificial network containing 130 species and 130 reaction rates, using (**A**) noiseless and (**B**) 20%-noise data. Asterisks (*) denote the top methods with respect to accuracy. Pluses (+) denote the top methods with respect to runtime. When several methods have the same score, it often means these methods reached the same local minimum. Multiple scoring schemes (species MRE, PPE and weighted SSE) were used to compare the parameter estimation quality, but we advocate species MRE, as it specifies the maximum distance between the simulated and observed trajectories for all species. Each point was perturbed with noise according to a Gaussian distribution with zero mean, and with standard deviation equal to the specified percent of the nominal data value.

A

		Noise = 0												
	#species	30	40	50	60	70	80	90	100	110	120	130	140	150
	#rates	30	40	50	60	70	80	90	100	110	120	130	140	150
Run time (s)	SPEDRE-base	179.00	371.00	403.00	438.00	445.00	820.00	1074.00	967.00	1296.00	1361.00	1313.00	2030.00	1523.00
	SD	45.85	81.76	11.68	314.40	392.83	191.30	1620.82	2170.96	3419.98	2347.64	7703.70	11940.30	933.06
	SRES	58.80	92.84	156.70	270.27	316.99	611.26	1021.93	1160.46	1480.59	2598.70	3479.96	5057.88	5062.28
	PSO	41.12	71.39	113.57	178.25	212.99	484.20	656.73	961.04	983.49	1027.77	3261.39	2871.87	3390.76
	LM	6.08	11.82	26.49	43.66	54.85	264.25	361.56	2378.30	742.78	7869.39	8861.93	7311.95	13759.10
	GA	11.59	16.22	31.59	46.88	54.13	116.08	171.21	177.17	265.93	519.84	365.74	369.99	942.00
	SPEDRE	184.90	383.50	428.66	482.30	497.90	1094.14	1341.31	2378.14	1837.57	2846.32	3382.29	3922.84	4590.40
	SRES_LM	65.63	104.22	188.65	314.28	361.84	844.50	1298.83	1721.88	2237.46	6142.50	5363.98	6954.63	6959.03
	PSO_LM	46.71	84.24	140.32	223.41	258.93	719.27	924.70	1494.72	1495.69	2405.80	5268.79	5170.96	10030.72
	GA_LM	18.30	27.19	59.00	114.24	106.81	375.81	480.83	1547.64	774.23	1745.40	4726.79	3056.65	3935.97
	SPEDRE-base_SD	199.22	376.69	491.70	593.56	661.64	1332.56	1870.68	1370.84	2936.21	2794.18	6406.89	5034.06	3277.99
	SRES_SD	78.80	98.90	163.54	432.76	504.49	1053.32	1853.88	2166.70	3110.46	5773.13	3827.50	10126.94	12500.08
	PSO_SD	57.28	104.94	140.76	353.53	410.83	846.24	1538.01	2156.44	2538.13	3021.81	4624.04	7522.93	5931.39
	GA_SD	15.91	47.11	125.78	109.57	76.10	283.73	200.20	262.58	414.52	832.31	2470.91	606.38	1870.73
Species MRE	SPEDRE-base	1.55	0.14	0.54	0.51	1.07	1.73	3.08	3.51	3.00	3.28	5.59	1.82	3.51
	SD	1.90	0.01	2.50	0.63	0.18	1.77	1.85	1.44	3.95	3.74	1.43	1.51	4.32
	SRES	0.14	0.07	0.43	0.07	0.21	0.38	1.92	1.82	2.33	1.75	2.03	1.59	1.47
	PSO	0.21	0.00	0.13	0.04	0.10	0.10	0.16	1.37	1.39	2.28	1.66	1.34	1.97
	LM	0.00	0.00	0.00	0.00	0.00	0.00	0.00	2.18	0.00	2.23	2.48	2.39	2.19
	GA	0.32	0.06	0.35	2.01	1.24	0.69	1.93	3.34	2.38	4.74	5.02	4.65	2.99
	SPEDRE	0.00	0.00	0.00	0.00	0.00	0.00	0.00	0.13	0.00	0.00	0.00	0.00	0.12
	SRES_LM	0.00	0.00	0.00	0.00	0.00	0.00	0.00	0.00	0.00	0.03	0.00	0.00	0.00
	PSO_LM	0.00	0.00	0.00	0.00	0.00	0.00	0.00	0.00	0.00	0.00	0.00	0.00	0.13
	GA_LM	0.00	0.00	0.00	0.00	0.00	0.00	0.00	0.10	0.00	0.00	2.48	0.00	0.00
	SPEDRE-base_SD	0.09	0.09	0.17	0.06	0.08	0.21	0.18	2.27	1.60	1.53	1.01	0.78	2.27
	SRES_SD	0.11	0.06	0.43	0.06	0.06	0.36	1.93	1.37	2.35	0.93	1.95	1.29	1.38
	PSO_SD	0.19	0.00	0.13	0.04	0.10	0.09	0.16	1.30	1.39	1.64	1.67	1.03	1.85
	GA_SD	0.24	0.03	0.35	0.47	1.18	0.44	1.93	3.34	2.38	4.34	5.02	4.65	2.87
Median PPE	SPEDRE-base	27.48	9.46	14.35	11.46	16.87	14.90	13.59	24.68	23.04	20.36	14.19	12.57	18.07
	SD	32.54	4.20	67.69	23.81	29.29	42.41	59.20	28.70	52.41	45.93	28.26	27.87	65.22
	SRES	18.66	13.53	38.71	29.67	32.58	29.69	28.60	32.68	30.55	34.69	47.00	42.75	35.73
	PSO	8.95	6.29	11.04	22.17	16.29	18.28	31.39	33.23	20.21	32.37	41.24	34.79	36.50
	LM	0.00	0.00	0.00	0.00	0.00	0.00	0.00	9.46	0.00	8.54	3.66	2.94	8.00
	GA	28.36	26.15	42.18	75.22	65.25	48.71	48.29	80.16	35.85	68.05	80.80	85.08	59.39
	SPEDRE	0.00	0.00	0.00	0.00	0.00	0.00	0.00	1.53	0.00	0.00	0.00	0.00	1.16
	SRES_LM	0.00	0.00	0.00	0.00	0.00	0.00	0.00	0.00	0.00	0.32	0.00	0.00	0.00
	PSO_LM	0.00	0.00	0.00	0.00	0.00	0.00	0.00	0.00	0.00	0.00	0.00	0.00	1.23
	GA_LM	0.00	0.00	0.00	0.00	0.00	0.00	0.00	1.20	0.00	0.00	4.11	0.00	0.00
	SPEDRE-base_SD	15.00	8.99	20.63	7.54	12.58	11.93	11.91	25.76	20.18	19.25	9.10	11.22	17.48
	SRES_SD	17.54	12.90	38.69	27.41	24.57	29.15	27.77	23.80	26.69	20.23	46.97	35.11	24.46
	PSO_SD	6.82	4.04	11.07	20.53	14.76	17.35	31.07	28.79	19.64	22.92	40.59	29.54	35.21
	GA_SD	23.91	7.17	38.74	68.82	65.22	42.51	48.29	80.16	34.93	65.84	79.98	85.08	59.17
Maximum PPE	SPEDRE-base	237.38	471.52	872.72	94.60	519.65	3176.70	2101.73	1322.18	3436.00	1078.67	1078.67	1322.18	1322.18
	SD	625.76	2659.10	8329.70	670.23	5888.93	20566.17	3978.75	8772.20	17238.52	2460.96	3030.34	1469.82	6602.07
	SRES	118.88	877.92	629.13	531.54	4382.51	2381.09	1595.55	1948.94	17032.30	5740.76	4490.07	9452.44	1944.64
	PSO	183.45	916.74	995.66	113.44	195.62	2860.01	2439.33	1443.32	5924.59	10551.76	5279.34	880.36	3382.48
	LM	0.00	0.21	0.01	0.00	0.02	0.09	0.02	2457.33	0.01	2131.87	375.86	472.85	2341.52
	GA	298.17	965.15	867.51	269.92	909.93	3170.23	5041.65	2926.78	9206.24	3599.76	7302.22	2269.59	9361.82
	SPEDRE	0.00	0.21	0.01	0.00	0.02	0.09	0.02	120.54	0.01	0.02	0.00	0.02	114.07
	SRES_LM	0.00	0.21	0.01	0.00	0.02	0.09	0.02	0.01	0.01	64.12	0.00	0.02	0.01
	PSO_LM	0.00	0.21	0.01	0.00	0.02	0.09	0.02	0.01	0.01	0.02	0.00	0.02	168.41
	GA_LM	0.00	0.21	0.01	0.00	0.02	0.10	0.02	104.02	0.01	0.02	369.81	0.02	0.01
	SPEDRE-base_SD	163.46	459.39	632.60	89.58	407.41	508.05	2102.31	1330.38	3486.13	696.18	767.21	1304.98	1330.38
	SRES_SD	110.79	877.72	629.14	527.14	3934.94	2359.20	1564.14	1262.06	16963.00	4731.55	4485.81	6973.70	1882.32
	PSO_SD	155.06	881.96	995.78	110.93	188.06	2859.58	2439.40	1169.56	5906.41	6398.32	5271.96	841.59	3282.58
	GA_SD	297.65	1045.57	845.51	266.91	910.23	3113.74	5041.65	2926.78	9206.24	3586.45	7262.57	2269.60	9320.24
Weighted SSE	SPEDRE-base	23.13	5.03	18.80	3.90	36.87	26.27	29.50	480.92	40.72	65.07	71.35	62.15	495.80
	SD	9.58	0.06	53.89	6.34	3.76	44.35	66.39	15.30	25.57	29.95	23.12	12.87	359.18
	SRES	1.46	0.49	3.62	2.24	4.72	7.66	23.39	20.74	10.50	18.10	35.88	29.20	40.59
	PSO	1.02	0.05	0.73	1.17	1.48	2.43	4.77	7.39	7.56	12.88	19.05	14.76	31.28
	LM	0.00	0.00	0.00	0.00	0.00	0.00	0.00	19.83	0.00	22.31	8.07	10.51	19.25
	GA	6.86	1.59	7.65	57.48	45.11	23.28	63.96	206.90	21.51	91.17	291.94	296.91	95.13
	SPEDRE	0.00	0.00	0.00	0.00	0.00	0.00	0.00	0.02	0.00	0.00	0.00	0.00	0.02
	SRES_LM	0.00	0.00	0.00	0.00	0.00	0.00	0.00	0.00	0.00	0.00	0.00	0.00	0.00
	PSO_LM	0.00	0.00	0.00	0.00	0.00	0.00	0.00	0.00	0.00	0.00	0.00	0.00	0.05
	GA_LM	0.00	0.00	0.00	0.00	0.00	0.00	0.00	0.02	0.00	0.00	8.05	0.00	0.00
	SPEDRE-base_SD	2.06	2.79	4.50	0.54	1.81	2.13	3.75	32.40	13.20	14.41	2.79	3.41	35.58
	SRES_SD	1.02	0.43	3.61	1.86	2.25	6.52	20.15	9.61	6.93	4.53	35.42	20.18	12.16
	PSO_SD	0.84	0.02	0.69	0.91	1.03	2.24	4.58	5.81	6.63	8.86	17.40	10.58	29.05
	GA_SD	5.81	0.18	5.04	19.74	40.63	10.10	63.96	206.67	20.32	44.23	236.46	295.48	83.22

B

	Noise = 20%													
	#species	30	40	50	60	70	80	90	100	110	120	130	140	150
	#rates	30	40	50	60	70	80	90	100	110	120	130	140	150
Run time (s)	SPEDRE-base	218.00	337.00	394.00	476.00	623.00	854.00	663.00	978.00	1334.00	1526.00	1064.00	971.00	1131.00
	SD	46.18	89.19	11.64	316.70	514.65	257.48	1599.03	2125.53	3167.19	3153.12	7448.58	3874.49	933.42
	SRES	67.91	100.85	162.09	266.29	366.18	1022.04	910.20	1105.80	1420.47	2417.06	3434.50	4284.40	4967.57
	PSO	38.16	75.86	111.68	175.06	291.10	561.95	637.14	924.54	966.24	1587.53	2420.28	2422.15	3296.85
	LM	33.43	70.15	168.29	274.05	364.45	689.68	960.56	1165.86	2993.21	6320.72	9437.22	7686.98	13845.50
	GA	11.01	18.17	28.41	38.95	81.31	175.38	151.01	152.66	240.05	360.74	623.43	696.54	900.67
	SPEDRE	451.19	713.20	868.42	1093.52	1486.02	2295.08	1823.81	3370.43	4082.31	5944.94	6351.52	5904.16	7852.76
	SRES_LM	83.07	135.02	241.65	408.60	610.45	1476.64	1444.57	2470.96	2800.64	5257.76	7769.19	8540.23	12118.42
	PSO_LM	54.60	111.42	188.78	315.36	529.44	1138.85	1265.09	2281.20	2382.06	4418.50	6690.32	6648.70	10082.04
	GA_LM	26.07	54.34	105.64	177.54	327.68	829.96	898.97	1094.38	1602.22	3288.61	4847.31	4999.45	7697.34
	SPEDRE-base_SD	236.30	384.55	489.41	641.28	878.14	1149.22	1338.42	2583.02	1665.05	2164.93	4774.39	4904.55	8001.94
	SRES_SD	88.39	144.35	171.96	430.30	645.24	1728.72	1770.72	1767.84	2682.67	4831.30	5502.14	5945.54	10262.09
	PSO_SD	56.33	79.69	116.52	328.93	570.18	614.44	1511.03	2194.90	2206.35	4633.73	6857.10	2572.82	4480.91
	GA_SD	27.80	64.58	51.98	160.38	119.89	279.41	877.63	179.88	447.78	1674.19	1133.25	1282.67	1931.93
Species MRE	SPEDRE-base	0.62	0.23	0.68	0.60	1.42	0.90	3.26	3.17	4.04	3.13	4.71	3.08	2.67
	SD	2.08	0.04	2.49	0.79	0.21	1.84	2.13	1.53	2.03	3.60	2.68	1.83	4.62
	SRES	0.25	0.09	0.15	0.14	0.21	0.50	1.85	1.58	2.99	1.78	2.12	1.66	2.01
	PSO	0.18	0.06	0.06	0.10	0.17	0.63	0.26	1.05	4.90	1.51	2.13	2.54	2.01
	LM	0.17	0.04	0.03	0.09	0.06	0.22	0.15	2.14	0.31	2.32	2.13	2.78	2.84
	GA	0.82	0.09	0.29	1.31	0.79	0.75	2.39	3.61	1.99	4.85	5.07	4.04	3.45
	SPEDRE	0.17	0.04	0.03	0.09	0.06	0.22	0.15	0.26	0.31	1.51	2.12	2.79	2.01
	SRES_LM	0.17	0.04	0.03	0.09	0.06	0.22	0.15	0.26	0.31	1.51	2.12	1.56	2.01
	PSO_LM	0.17	0.04	0.03	0.09	0.06	0.22	0.15	0.26	0.31	1.51	2.13	1.56	2.01
	GA_LM	0.17	0.04	0.03	0.09	0.06	0.22	0.15	2.15	0.31	2.31	2.13	1.56	2.01
	SPEDRE-base_SD	0.17	0.05	0.24	0.18	0.06	0.33	0.43	1.19	2.97	1.97	3.56	1.56	2.69
	SRES_SD	0.19	0.05	0.15	0.12	0.18	0.47	1.81	1.40	2.59	1.77	2.12	1.65	2.01
	PSO_SD	0.20	0.06	0.06	0.11	0.15	0.63	0.19	0.95	4.85	1.51	2.13	2.54	2.01
	GA_SD	0.34	0.05	0.24	0.30	0.77	0.68	1.97	3.61	2.00	3.98	4.29	3.89	2.93
Median PPE	SPEDRE-base	13.41	16.81	21.63	29.40	17.12	17.94	29.75	45.31	35.82	37.16	35.55	36.36	28.97
	SD	26.81	7.99	67.50	21.12	27.67	42.35	55.31	26.39	27.04	43.61	47.13	42.36	65.22
	SRES	14.24	21.62	45.60	24.40	26.05	24.04	35.09	32.22	31.92	28.97	42.76	43.67	34.29
	PSO	10.91	8.70	25.92	12.96	33.91	39.60	27.67	34.68	23.70	36.76	45.97	36.23	33.08
	LM	4.57	7.54	6.80	5.98	5.12	7.69	6.10	13.09	6.76	23.59	19.11	22.47	22.18
	GA	36.65	20.17	50.95	57.63	69.44	38.35	46.63	67.29	44.62	68.36	91.36	51.93	65.77
	SPEDRE	4.56	7.58	6.74	6.37	5.12	7.69	5.78	6.90	6.56	20.67	19.04	24.43	20.74
	SRES_LM	4.56	7.76	6.86	5.81	5.12	8.14	5.66	7.31	6.73	16.78	18.66	20.84	19.37
	PSO_LM	4.56	7.53	6.93	5.86	5.12	7.51	5.73	6.53	6.62	17.25	18.92	21.75	21.96
	GA_LM	4.56	7.47	6.87	6.18	5.12	7.88	5.92	11.86	6.65	22.56	19.70	21.45	20.37
	SPEDRE-base_SD	8.44	8.90	18.90	19.52	12.85	16.33	21.96	26.43	35.39	35.03	33.54	28.40	28.99
	SRES_SD	11.76	13.87	45.50	19.84	20.74	23.74	34.72	30.16	24.79	29.14	39.75	40.37	29.12
	PSO_SD	9.18	8.42	25.91	8.86	28.33	40.20	24.58	30.94	22.50	35.64	46.00	36.23	32.29
	GA_SD	28.57	11.59	49.60	44.78	69.23	35.31	42.83	67.29	44.61	66.60	89.71	51.65	65.76
Maximum PPE	SPEDRE-base	91.97	1614.55	446.12	94.60	3690.53	992.23	1375.99	1786.26	1078.67	3562.98	3941.98	2864.12	2325.19
	SD	614.91	2690.55	8321.16	1265.29	5918.13	20546.73	3940.45	7626.02	17028.11	2367.48	5390.70	4422.37	6602.05
	SRES	97.85	540.13	605.26	199.47	2347.42	1581.79	816.01	2747.54	14673.32	4694.30	1077.28	6786.43	2565.13
	PSO	121.47	193.26	1403.78	93.88	3378.73	4383.18	1517.32	833.99	6533.92	3055.62	2910.83	2016.52	12826.11
	LM	31.73	2018.79	1426.18	54.84	91.58	617.49	2176.05	2356.21	120.80	2507.12	1058.79	1634.69	1273.24
	GA	368.26	1175.42	877.94	110.83	2333.11	5992.86	2982.71	3866.59	558.68	3210.93	935.67	2857.30	18994.64
	SPEDRE	32.97	2031.75	1426.18	55.21	91.58	667.07	2177.95	795.87	316.41	322.57	1063.96	1639.72	964.11
	SRES_LM	33.10	2061.66	1426.18	54.83	91.58	797.79	2201.72	698.90	115.77	370.60	1083.64	970.82	1058.46
	PSO_LM	32.97	2025.21	1426.18	55.48	91.58	601.37	2251.30	761.80	99.83	1049.41	1073.30	1012.75	964.79
	GA_LM	32.91	2000.01	1426.18	54.26	91.58	674.29	2188.84	2369.24	203.50	2416.73	1175.29	866.83	1006.20
	SPEDRE-base_SD	73.69	1567.19	93.98	93.80	3094.34	975.27	808.96	1737.04	1092.61	3577.90	3940.50	2498.67	2312.60
	SRES_SD	87.34	726.95	604.68	189.53	2164.01	1581.41	740.72	2641.92	14371.91	4205.20	1083.35	5995.12	2170.82
	PSO_SD	113.66	192.93	1403.78	93.03	3275.05	4383.32	1516.37	598.21	6517.54	2847.95	2910.79	2016.52	12727.29
	GA_SD	354.57	1238.28	878.42	108.00	2333.10	5989.61	2417.63	3866.59	544.95	3208.83	858.57	2797.69	18993.98
Weighted SSE	SPEDRE-base	8.60	4.68	16.63	18.32	36.53	16.99	51.56	577.58	60.17	1721.13	1354.80	5819.39	610.46
	SD	9.58	1.16	54.33	8.52	5.99	47.48	62.54	27.09	9.74	172.17	289.92	234.15	512.19
	SRES	3.33	1.64	5.49	4.72	5.41	7.98	19.22	25.43	19.86	161.01	275.56	230.12	122.01
	PSO	1.50	1.29	1.52	2.35	5.34	24.25	8.50	21.83	11.81	159.34	264.46	222.68	104.50
	LM	1.28	1.06	1.05	1.86	1.82	2.59	2.83	31.71	3.17	161.24	235.46	208.96	105.18
	GA	14.66	2.34	6.92	24.72	36.26	21.44	38.21	145.10	21.83	266.69	615.43	276.80	202.57
	SPEDRE	1.28	1.06	1.05	1.86	1.82	2.59	2.83	12.47	3.17	146.49	235.72	210.69	78.98
	SRES_LM	1.28	1.06	1.05	1.86	1.82	2.60	2.83	12.52	3.17	146.64	235.16	200.31	78.83
	PSO_LM	1.28	1.06	1.05	1.86	1.82	2.59	2.83	12.47	3.17	146.40	234.92	200.26	78.99
	GA_LM	1.28	1.07	1.05	1.86	1.82	2.60	2.83	31.67	3.17	161.29	235.80	200.33	78.86
	SPEDRE-base_SD	2.43	1.16	4.17	4.60	2.71	8.80	9.49	19.12	40.38	184.38	343.15	227.37	189.19
	SRES_SD	2.83	1.27	5.44	3.25	4.28	7.52	18.42	23.27	12.59	158.30	270.34	224.91	106.56
	PSO_SD	1.42	1.28	1.51	2.06	4.11	23.86	7.11	17.54	26.96	156.71	263.99	222.68	102.94
	GA_SD	6.32	1.18	6.38	8.65	33.05	17.94	23.91	145.10	20.83	252.69	508.89	272.44	192.69

Table S7 - Comparison of standalone and hybrid methods on artificial networks of size N=30 to 150, using (**A**) noiseless and (**B**) 20%-noise dataset. Each dataset used in each test case was constructed by simulating the network using nominal reaction rates, where no noise or 20%-noise (Gaussian) was added to the data. Multiple scoring schemes (species MRE, PPE and weighted SSE) were used to compare the parameter estimation accuracy. Cells highlighted in reds have the best score among all methods being compared for each test case (*i.e.* column).

A

		Run time				Species MRE				Normalized SSE			
	**Species**	120	130	140	150	120	130	140	150	120	130	140	150
	**Rates**	120	130	140	150	120	130	140	150	120	130	140	150
Noise=5%	**SPEDRE-base**	123.05±13.39	123.51±5.37	128.63±8.61	134.57±7.91	0.87±0.16	1.14±0.30	1.33±0.74	1.11±0.12	3.57±0.44	4.93±2.17	5.40±1.53	5.99±1.50
	**SD**	5.93±0.78	6.98±1.00	28.89±21.12	10.77±2.66	1.90±0.89	2.31±1.24	0.92±0.66	2.05±0.98	14.99±4.12	23.03±12.66	9.99±14.08	24.40±15.13
	**SRES**	38.67±0.84	42.41±2.15	49.68±3.33	51.60±5.77	0.61±0.18	0.83±0.15	0.79±0.22	0.96±0.75	2.84±0.31	2.59±0.32	3.15±0.51	4.26±1.49
	**PSO**	26.70±1.31	31.68±2.89	36.55±2.33	37.36±5.50	0.72±0.13	0.91±0.52	1.07±0.66	1.24±0.37	2.10±0.67	3.16±0.36	2.70±0.49	4.74±0.46
	**LM**	76.71±2.57	96.35±2.02	123.89±7.82	143.57±7.49	0.20±0.02	0.21±0.03	0.19±0.03	0.21±0.03	0.50±0.03	0.54±0.01	0.61±0.06	0.67±0.02
	**GA**	6.66±0.64	7.31±0.67	8.44±0.55	8.99±0.54	1.27±0.17	1.46±0.14	1.50±0.25	1.43±0.15	10.46±1.10	7.72±0.39	14.39±1.66	11.52±1.13
	**SPEDRE**	162.22±13.89	172.52±6.53	192.16±8.18	205.98±5.48	0.20±0.02	0.21±0.03	0.19±0.03	0.21±0.03	0.50±0.03	0.54±0.01	0.62±0.06	0.67±0.02
	**SRES_LM**	78.33±0.87	91.63±2.03	112.32±4.70	122.78±8.68	0.20±0.02	0.21±0.03	0.19±0.03	0.21±0.03	0.50±0.03	0.54±0.01	0.61±0.06	0.67±0.02
	**PSO_LM**	65.96±2.30	81.14±3.95	99.90±4.69	108.88±9.39	0.20±0.02	0.21±0.03	0.19±0.03	0.21±0.03	0.50±0.03	0.54±0.01	0.61±0.06	0.67±0.02
	**GA_LM**	46.11±0.22	55.63±0.83	70.27±5.76	78.79±3.48	0.20±0.02	0.21±0.03	0.19±0.03	0.21±0.03	0.50±0.03	0.54±0.01	0.62±0.06	0.67±0.02
	**SPEDRE-base_SD**	140.47±11.31	133.16±10.37	162.56±21.63	168.87±36.53	0.43±0.17	0.98±0.31	0.61±0.48	0.68±0.30	1.17±0.82	3.11±1.99	1.59±0.73	1.78±0.99
	**SRES_SD**	70.04±24.27	47.81±2.59	78.20±23.99	80.24±43.76	0.51±0.30	0.85±0.10	0.58±0.25	0.89±0.81	1.45±1.23	2.41±0.40	2.15±1.26	3.31±2.55
	**PSO_SD**	58.00±23.64	35.58±1.43	43.75±3.95	45.26±7.42	0.56±0.29	0.91±0.53	1.05±0.67	1.20±0.30	1.12±0.29	3.08±0.50	2.42±0.35	4.51±0.62
	**GA_SD**	10.30±1.32	11.71±1.18	14.80±1.45	13.30±0.94	1.23±0.22	1.42±0.13	1.47±0.23	1.42±0.15	9.93±1.83	7.57±0.46	13.46±2.94	11.46±1.06
Noise=10%	**SPEDRE-base**	119.12±6.69	122.47±5.32	129.75±7.92	134.84±6.03	0.96±0.13	1.17±0.20	1.08±0.10	1.07±0.21	7.74±1.51	9.72±3.27	10.06±1.69	10.85±2.21
	**SD**	5.71±0.88	7.04±0.87	26.56±16.96	10.96±2.69	1.94±0.81	2.34±1.27	1.01±0.69	2.11±1.03	16.73±3.56	25.23±12.88	12.45±14.09	28.57±15.12
	**SRES**	36.20±1.20	41.37±5.58	49.50±3.50	49.81±8.06	0.74±0.16	0.78±0.20	0.86±0.33	0.85±0.15	3.98±0.73	4.59±0.58	5.15±0.28	6.85±0.95
	**PSO**	27.33±1.52	32.64±2.69	38.44±0.96	37.51±4.53	0.97±0.29	0.93±0.20	0.76±0.13	1.14±0.14	4.88±2.04	6.05±0.56	5.24±0.97	7.16±0.97
	**LM**	76.65±5.58	95.96±2.99	125.96±4.03	139.64±8.81	0.44±0.05	0.44±0.09	0.41±0.11	0.46±0.07	2.02±0.12	2.20±0.02	2.50±0.24	2.73±0.07
	**GA**	6.68±0.83	7.31±0.13	8.06±0.16	9.65±0.55	1.17±0.17	1.47±0.32	1.58±0.03	1.41±0.26	10.32±0.51	11.42±2.87	16.46±3.65	14.06±0.86
	**SPEDRE**	157.74±8.75	171.02±7.76	192.64±7.39	206.00±5.07	0.44±0.05	0.44±0.09	0.41±0.11	0.46±0.07	2.03±0.12	2.20±0.02	2.50±0.24	2.73±0.08
	**SRES_LM**	75.08±1.66	90.15±3.96	113.01±1.41	120.19±11.22	0.44±0.05	0.44±0.09	0.41±0.11	0.46±0.07	2.03±0.12	2.20±0.02	2.50±0.25	2.73±0.07
	**PSO_LM**	65.88±3.04	81.28±3.79	102.69±3.81	108.66±6.94	0.44±0.05	0.44±0.09	0.41±0.11	0.46±0.07	2.03±0.12	2.20±0.02	2.50±0.24	2.73±0.08
	**GA_LM**	45.72±1.35	56.05±1.60	70.15±4.04	79.21±2.37	0.44±0.05	0.44±0.09	0.41±0.11	0.46±0.07	2.03±0.12	2.20±0.02	2.50±0.24	2.73±0.07
	**SPEDRE-base_SD**	136.76±19.95	129.10±5.52	174.58±23.00	153.81±20.09	0.60±0.18	0.93±0.26	0.50±0.04	0.72±0.32	4.34±2.48	7.46±3.28	3.75±0.95	5.72±1.79
	**SRES_SD**	53.19±25.02	63.43±34.16	61.31±7.68	56.55±10.16	0.67±0.25	0.72±0.29	0.74±0.18	0.83±0.16	3.39±1.11	4.08±1.09	4.39±0.78	6.56±1.06
	**PSO_SD**	30.54±2.74	35.71±4.15	72.85±36.44	45.79±2.40	0.97±0.29	0.93±0.20	0.59±0.06	0.95±0.14	4.85±2.05	6.04±0.57	4.40±1.63	6.47±1.65
	**GA_SD**	12.69±4.21	12.42±1.29	21.15±13.23	20.18±7.50	0.95±0.21	1.38±0.30	1.26±0.47	1.15±0.54	7.59±3.47	10.19±3.02	13.14±7.86	10.97±5.07
Noise=20%	**SPEDRE-base**	117.61±6.20	122.58±4.80	128.44±8.57	134.76±7.76	2.13±1.09	1.68±0.45	2.15±0.72	1.54±0.06	24.27±7.84	21.38±4.06	25.90±1.91	28.12±2.00
	**SD**	5.71±0.78	6.31±0.62	9.27±1.31	10.29±1.91	2.15±0.73	2.68±1.48	1.88±0.38	2.32±0.95	24.48±3.12	34.99±11.87	30.97±9.14	41.55±14.84
	**SRES**	38.40±3.48	41.41±6.84	50.61±5.83	49.75±7.86	1.09±0.22	1.14±0.03	1.13±0.34	1.21±0.11	11.12±1.18	12.47±0.35	13.81±1.73	15.79±1.73
	**PSO**	26.07±2.26	30.12±1.99	34.76±2.61	39.06±2.03	1.06±0.23	1.11±0.05	1.00±0.41	1.21±0.11	9.93±0.62	11.81±1.89	13.75±3.27	15.18±0.27
	**LM**	74.10±2.36	95.89±4.89	123.76±4.29	139.60±3.86	1.06±0.23	1.02±0.21	1.00±0.41	1.15±0.14	8.40±0.41	9.03±0.02	10.26±1.14	11.19±0.31
	**GA**	6.49±0.61	7.22±0.63	8.55±0.22	9.77±0.60	1.53±0.40	1.22±0.14	1.44±0.23	1.37±0.05	18.22±0.52	18.24±2.08	24.95±1.11	22.42±0.67
	**SPEDRE**	155.60±6.29	171.58±6.95	190.52±10.47	205.71±6.40	1.06±0.23	1.02±0.21	1.00±0.41	1.15±0.14	8.40±0.41	9.03±0.02	10.27±1.14	11.19±0.31
	**SRES_LM**	76.77±3.81	89.72±5.21	111.58±4.98	120.28±9.40	1.06±0.23	1.02±0.21	1.00±0.41	1.15±0.14	8.41±0.41	9.03±0.02	10.27±1.14	11.20±0.31
	**PSO_LM**	64.11±3.34	78.09±4.09	96.31±0.93	110.21±5.17	1.06±0.23	1.02±0.21	1.00±0.41	1.15±0.14	8.41±0.41	9.03±0.02	10.27±1.14	11.20±0.31
	**GA_LM**	44.74±1.33	54.69±1.61	68.86±2.81	79.05±3.13	1.06±0.23	1.02±0.21	1.00±0.41	1.15±0.14	8.40±0.41	9.03±0.02	10.27±1.14	11.20±0.31
	**SPEDRE-base_SD**	132.20±10.62	128.89±5.22	150.65±22.08	145.32±8.24	1.24±0.23	1.46±0.26	1.26±0.53	1.27±0.18	13.24±3.83	17.68±4.66	16.95±4.82	21.68±3.28
	**SRES_SD**	43.08±1.70	46.12±8.18	60.82±12.09	59.84±12.39	1.08±0.22	1.09±0.10	1.07±0.36	1.21±0.11	10.79±1.67	12.30±0.36	13.32±1.73	14.89±2.46
	**PSO_SD**	31.19±4.25	33.23±3.25	43.49±6.97	69.56±46.31	1.06±0.23	1.11±0.05	1.00±0.41	1.21±0.11	9.86±0.69	11.79±1.91	13.44±3.16	14.35±1.46
	**GA_SD**	9.86±1.30	11.23±1.91	13.52±2.42	16.43±2.24	1.30±0.04	1.08±0.11	1.37±0.24	1.37±0.05	16.60±1.94	16.62±0.29	24.05±1.58	20.55±3.72

B

		Median PPE				Maximum PPE			
	Species	120	130	140	150	120	130	140	150
	Rates	120	130	140	150	120	130	140	150
Noise=5%	SPEDRE-base	28.83±3.02	29.56±2.40	23.57±1.60	25.65±4.55	9893.26±8011.82	6994.62±7147.31	2903.01±2503.68	8673.30±11821.01
	SD	48.73±3.96	54.83±11.61	30.69±14.80	51.06±12.39	10006.10±5170.89	8638.17±4859.87	6463.19±4402.95	9244.00±8266.04
	SRES	27.63±2.37	25.69±2.09	25.64±3.34	33.39±9.75	5079.13±1528.58	6306.72±5444.89	6184.20±4628.57	4971.46±3694.82
	PSO	24.81±0.91	28.12±3.34	21.95±1.41	28.14±4.91	7219.21±5530.18	7010.67±6532.88	16608.83±11513.38	9730.38±5588.08
	LM	10.18±2.28	9.89±1.17	8.75±0.64	9.54±1.12	3691.49±3548.48	1671.74±1343.94	4511.92±6083.07	1382.85±1987.82
	GA	50.82±7.56	9.89±1.18	43.35±2.75	48.25±5.10	4834.38±2661.68	8106.47±6651.68	7370.11±5491.02	3133.13±712.82
	SPEDRE	9.96±1.95	9.89±1.19	8.87±0.78	8.97±0.72	3365.06±3078.74	1715.61±1323.76	4479.17±5981.37	1441.09±1924.94
	SRES_LM	10.45±2.58	9.89±1.20	8.93±0.85	9.22±1.17	3327.66±3035.00	1668.23±1341.68	4487.11±6069.66	1398.88±2005.37
	PSO_LM	10.17±2.08	9.89±1.21	8.97±0.95	9.30±1.10	4714.68±5128.85	1709.14±1323.20	4519.74±6093.00	1374.99±1979.80
	GA_LM	10.42±2.43	9.89±1.22	8.80±0.96	9.48±0.52	3518.81±3285.96	1703.65±1365.95	4487.18±6034.87	1369.69±1989.14
	SPEDRE-base_SD	16.48±5.95	9.89±1.23	16.26±3.43	16.58±2.11	9290.23±7999.02	6951.27±7209.76	3275.32±3117.58	8457.97±12031.11
	SRES_SD	17.82±8.43	25.31±1.90	20.07±7.51	27.80±17.60	4859.03±1148.69	6114.15±5422.69	6212.19±4936.13	5193.32±4095.22
	PSO_SD	19.33±4.35	27.94±3.22	20.42±1.55	27.54±5.77	5161.47±3063.44	7013.22±6530.86	16541.54±11457.04	9727.14±5593.02
	GA_SD	50.66±7.76	49.03±5.86	43.33±2.79	48.21±5.13	4821.70±2655.23	8108.59±6650.26	7341.31±5524.31	3134.38±711.16
Noise=10%	SPEDRE-base	32.76±7.63	33.93±6.37	28.47±1.33	37.05±4.66	11677.89±8265.73	4333.46±3456.99	5115.96±3424.02	5800.83±6228.43
	SD	48.88±3.51	55.30±11.66	33.47±12.54	52.81±10.73	9945.96±5073.32	8653.17±4847.69	6620.86±4627.86	9259.57±8237.45
	SRES	28.03±4.57	29.84±4.36	26.15±0.63	31.17±7.74	9609.72±8655.88	4023.88±2750.89	7552.71±3949.99	5803.94±4930.90
	PSO	24.63±5.17	29.67±1.32	28.18±3.80	31.50±2.60	12608.83±9284.06	10028.80±8176.51	8831.73±11846.71	5553.94±2636.63
	LM	18.08±3.33	16.85±2.55	16.04±1.07	15.70±1.08	3755.43±4020.20	2226.04±1824.66	8547.28±12945.94	2727.58±4002.30
	GA	51.55±7.84	50.40±8.63	49.55±10.22	48.59±2.58	9210.91±10712.42	2931.04±1718.84	6149.93±3955.00	7295.62±5962.50
	SPEDRE	18.17±3.06	17.23±2.54	16.00±1.12	15.45±0.66	3512.32±4353.73	2265.46±1868.91	8543.88±12949.17	2801.73±3887.38
	SRES_LM	18.08±2.59	16.35±1.74	16.31±0.84	15.70±0.89	3141.75±4531.42	2143.70±1817.92	8550.54±12942.85	2845.51±3911.58
	PSO_LM	17.87±2.69	16.76±2.24	16.26±1.05	16.20±1.73	3034.10±4336.70	2148.01±1799.93	8538.95±12953.84	2767.59±3980.39
	GA_LM	18.93±3.76	16.43±2.23	16.01±1.12	16.34±1.04	3725.84±4114.87	2303.72±1941.48	8540.88±12952.00	2756.50±3926.54
	SPEDRE-base_SD	24.58±12.15	30.45±7.40	17.60±2.28	29.45±5.38	10070.22±7798.73	4572.03±3406.38	5838.55±4486.23	5769.77±6276.92
	SRES_SD	23.57±5.98	27.43±3.68	23.69±1.07	30.77±7.58	7721.43±5405.14	4151.46±2462.46	7502.95±4110.71	5802.75±4938.04
	PSO_SD	24.59±5.16	29.68±1.34	24.14±2.75	31.51±2.87	12608.24±9284.37	10028.61±8176.67	8754.97±11914.78	5302.93±3067.45
	GA_SD	46.70±12.98	48.40±10.35	45.34±19.29	44.25±9.10	9292.45±10665.78	2896.40±1662.87	5933.11±4314.53	7333.71±6053.00
Noise=20%	SPEDRE-base	49.24±5.05	39.17±4.24	39.62±2.68	46.90±4.95	6579.11±5932.49	5671.30±4290.22	5758.56±6168.89	4719.87±1196.02
	SD	48.57±2.43	56.12±10.70	45.85±6.37	54.22±9.21	9997.51±5178.93	8886.13±4591.03	6553.39±3254.59	9263.56±8196.07
	SRES	31.41±0.52	33.38±3.73	30.62±4.75	36.21±10.76	10321.80±8101.72	4653.39±4019.86	10968.90±6975.67	4520.43±2920.41
	PSO	30.22±5.44	38.65±14.42	32.05±10.18	35.37±1.48	12466.17±10150.54	9181.65±12459.36	9736.66±11003.49	10945.12±11219.87
	LM	30.57±4.65	28.87±4.99	28.46±3.83	24.70±1.03	7489.46±11187.72	2963.98±3089.12	8662.95±12837.48	2964.70±4096.84
	GA	49.64±3.36	44.68±3.47	50.49±3.75	47.04±2.92	4313.58±3985.72	6733.28±6171.02	4214.61±1821.21	4539.66±2118.29
	SPEDRE	31.28±4.57	28.88±5.46	27.95±4.11	25.11±0.38	7644.14±11451.73	3044.00±3227.03	8661.72±12838.62	3005.07±4057.93
	SRES_LM	30.41±4.40	27.09±4.23	28.29±3.79	25.26±0.14	7374.18±10986.60	2963.42±3088.58	8668.18±12832.62	2970.50±4091.17
	PSO_LM	30.99±4.09	29.03±5.93	27.10±3.34	25.86±1.25	7566.07±11330.73	2909.92±2966.89	8667.94±12832.85	2971.24±4090.33
	GA_LM	30.97±5.89	28.33±4.25	28.77±3.63	25.00±1.33	7736.27±11267.57	3472.60±3241.99	8661.52±12838.80	2956.35±4105.00
	SPEDRE-base_SD	38.21±2.40	35.98±7.34	34.07±5.80	43.67±3.39	6580.66±6233.20	5625.93±4364.53	5615.37±6420.95	4546.37±1421.17
	SRES_SD	29.32±3.76	33.62±4.06	29.66±6.80	36.38±10.73	10300.92±8114.16	4583.57±3926.44	10992.35±6997.97	4468.56±2860.64
	PSO_SD	29.23±4.89	38.64±14.43	31.60±9.23	32.58±4.63	12457.98±10142.99	9178.55±12461.48	9682.55±11038.70	10945.00±11219.88
	GA_SD	48.89±4.13	44.13±3.56	50.44±3.98	44.44±6.72	4302.24±4012.23	6582.08±6324.88	4196.82±1832.48	4558.66±2151.98

Table S8 - Comparison of standalone and hybrid rate parameter estimation methods applied to the artificial networks model. Methods were compared with respect to (**A**) runtime, species MRE, median PPE, (**B**) maximum PPE, and normalized SSE. Each cell in the table shows the average, together with standard deviation, of independent test results using different network structures (n=3). Each dataset used in each test case was constructed by simulating the network using nominal reaction rates. Gaussian noise (5%, 10%, and 20%) were added to the simulated dataset. The accuracy of each method was evaluated using species MRE, median PPE, maximum PPE, and weighted SSE. The best performing methods (for each column) were highlighted in red.

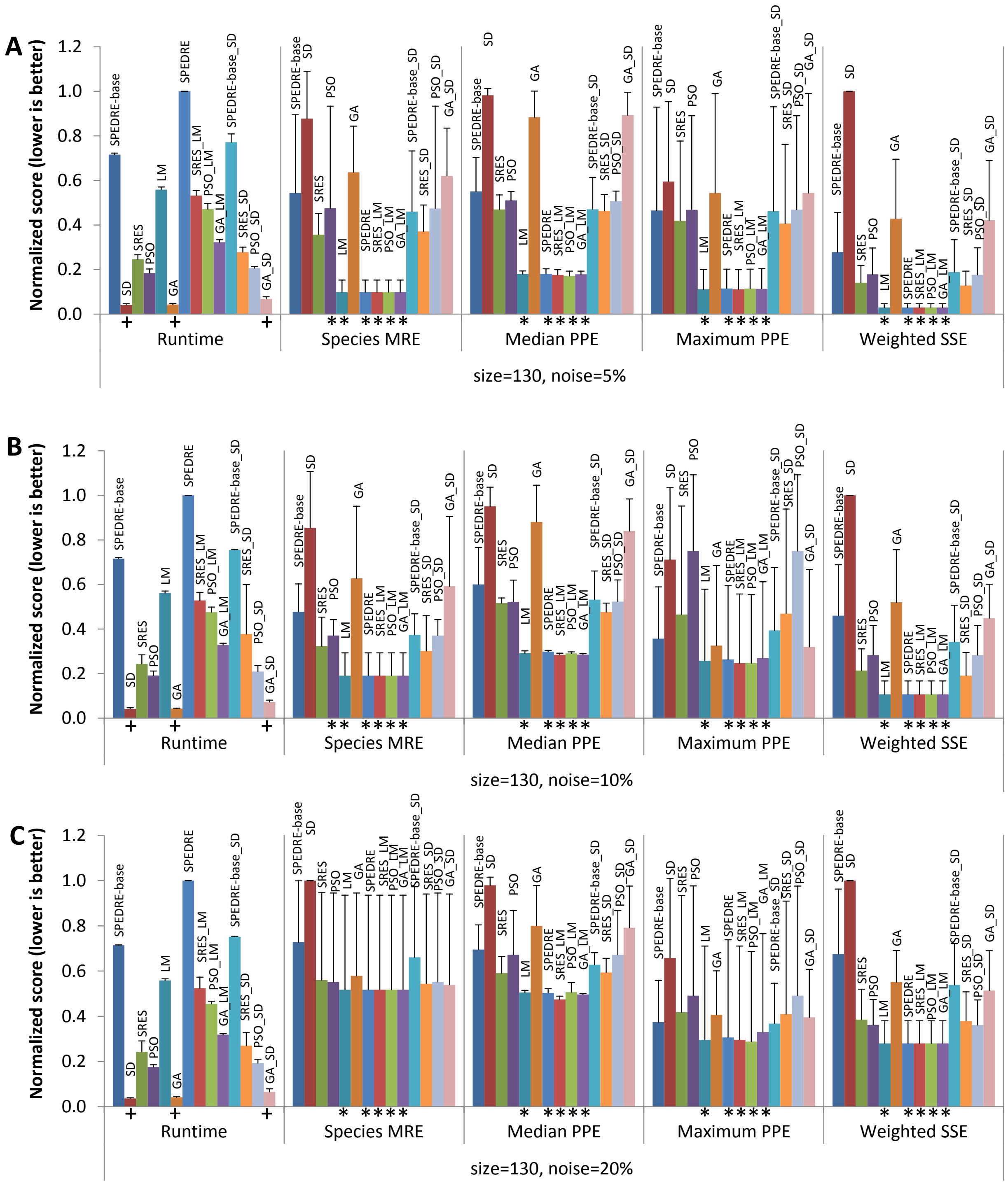

Figure S9 - Comparison of standalone and hybrid methods on artificial network containing 130 species and 130 reaction rates, using (**A**) 5%-noise, (B) 10%-noise, and (C) 20%-noise data. The columns represent the average results from 3 independent tests using different network structures, and error bars represent the standard deviation. Asterisks (*) denote the top methods with respect to accuracy. Pluses (+) denote the top methods with respect to runtime. When several methods have the same score, it often means these methods reached the same local minimum. Multiple scoring schemes (species MRE, PPE and weighted SSE) were used to compare the parameter estimation quality, but we advocate species MRE, as it specifies the maximum distance between the simulated and observed trajectories for all species.

A

	Species	Rates	Noise=5%		Noise=10%		Noise=20%	
			SPEDRE-base	SPEDRE-base-S	SPEDRE-base	SPEDRE-base-S	SPEDRE-base	SPEDRE-base-S
Run time	120	120	123.05±13.39	132.68±5.38	119.12±6.69	131.33±7.82	117.61±6.20	132.56±4.73
	130	130	123.51±5.37	135.84±4.93	122.47±5.32	136.03±4.82	122.58±4.80	136.99±5.45
	140	140	128.63±8.61	131.98±10.40	129.75±7.92	130.60±13.50	128.44±8.57	133.68±8.60
	150	150	134.57±7.91	135.41±21.36	134.84±6.03	136.68±19.28	134.76±7.76	135.80±21.19
Species MRE	120	120	0.87±0.16	2.32±0.95	0.96±0.13	5.07±3.00	2.13±1.09	4.08±1.60
	130	130	1.14±0.30	3.06±0.71	1.17±0.20	3.67±2.85	1.68±0.45	4.04±2.61
	140	140	1.33±0.74	4.26±0.61	1.08±0.10	4.16±0.61	2.15±0.72	3.54±1.04
	150	150	1.11±0.12	3.33±1.19	1.07±0.21	4.48±2.44	1.54±0.06	3.33±0.97
Median PPE	120	120	28.83±3.02	76.58±4.43	32.76±7.63	74.99±7.66	49.24±5.05	77.62±7.84
	130	130	29.56±2.40	75.00±6.85	33.93±6.37	75.90±5.10	39.17±4.24	78.03±7.09
	140	140	23.57±1.60	66.44±10.61	28.47±1.33	67.75±4.60	39.62±2.68	63.85±1.47
	150	150	25.65±4.55	80.85±4.99	37.05±4.66	78.48±6.78	46.90±4.95	71.66±8.31
Maximum PPE	120	120	9893.26±8011.82	7579.54±494.61	11677.89±8265.73	7464.81±2696.94	6579.11±5932.49	13365.55±7898.07
	130	130	6994.62±7147.31	11035.95±9758.01	4333.46±3456.99	14436.89±6805.04	5671.30±4290.22	14436.89±5932.49
	140	140	2903.01±2503.68	11293.78±9814.36	5115.96±3424.02	6017.05±863.61	5758.56±6168.89	9151.10±3216.63
	150	150	8673.30±11821.01	5572.44±1511.27	5800.83±6228.43	12579.77±8424.25	4719.87±1196.02	9436.66±1855.62
Normalized SSE	120	120	3.57±0.44	44.83±13.16	7.74±1.51	43.40±12.69	24.27±7.84	51.93±15.34
	130	130	4.93±2.17	45.21±4.91	9.72±3.27	45.75±14.85	21.38±4.06	58.65±7.83
	140	140	5.40±1.53	58.66±12.50	10.06±1.69	57.58±7.70	25.90±1.91	64.72±7.11
	150	150	5.99±1.50	65.71±12.68	10.85±2.21	59.31±12.48	28.12±2.00	61.02±4.83

B

	Species	Rates	Noise=5%		Noise=10%		Noise=20%	
			SPEDRE	SPEDRE-S	SPEDRE	SPEDRE-S	SPEDRE	SPEDRE-S
Run time	120	120	162.22±13.89	171.43±5.76	157.74±8.75	169.93±9.79	155.60±6.29	170.04±4.57
	130	130	172.52±6.53	182.84±3.86	171.02±7.76	182.13±5.27	171.58±6.95	183.01±5.68
	140	140	192.16±8.18	186.77±23.78	192.64±7.39	184.70±26.85	190.52±10.47	187.58±20.77
	150	150	205.98±5.48	194.09±39.11	206.00±5.07	196.79±37.11	205.71±6.40	194.92±37.69
Species MRE	120	120	0.20±0.02	0.20±0.02	0.44±0.05	0.44±0.05	1.06±0.23	1.06±0.23
	130	130	0.21±0.03	0.21±0.03	0.44±0.09	0.44±0.09	1.02±0.21	1.02±0.21
	140	140	0.19±0.03	0.19±0.03	0.41±0.11	0.41±0.11	1.00±0.41	1.00±0.41
	150	150	0.21±0.03	0.21±0.03	0.46±0.07	0.46±0.07	1.15±0.14	1.15±0.14
Median PPE	120	120	9.96±1.95	10.35±2.43	18.17±3.06	18.44±3.38	31.28±4.57	30.60±4.67
	130	130	9.86±0.84	9.89±1.28	17.23±2.54	17.01±2.37	28.88±5.46	28.00±4.78
	140	140	8.87±0.78	8.97±0.83	16.00±1.12	16.04±1.10	27.95±4.11	28.38±4.88
	150	150	8.97±0.72	9.73±0.98	15.45±0.66	15.78±0.66	25.11±0.38	25.19±0.79
Maximum PPE	120	120	3365.06±3078.74	3752.55±3611.36	3512.32±4353.73	3649.23±4059.92	7644.14±11451.73	7647.91±11456.51
	130	130	1715.61±1323.76	1698.98±1361.98	2265.46±1868.91	2214.69±1807.64	3044.00±3227.03	3120.64±3328.47
	140	140	4479.17±5981.37	4496.16±6052.59	8543.88±12949.17	8541.95±12950.99	8661.72±12838.62	8667.22±12833.51
	150	150	1441.09±1924.94	1429.57±2065.43	2801.73±3887.38	2828.42±4004.12	3005.07±4057.93	3008.07±4055.24
Normalized SSE	120	120	0.50±0.03	0.50±0.03	2.03±0.12	2.03±0.12	8.40±0.41	8.40±0.41
	130	130	0.54±0.01	0.54±0.01	2.20±0.02	2.20±0.02	9.03±0.02	9.03±0.03
	140	140	0.62±0.06	0.62±0.06	2.50±0.24	2.50±0.24	10.27±1.14	10.27±1.14
	150	150	0.67±0.02	0.67±0.02	2.73±0.08	2.73±0.07	11.19±0.31	11.19±0.31

Table S10. Performance comparison of (A) SPEDRE-base with cubic spline (SPEDRE-base) versus SPEDRE-base with smoothing spline (SPEDRE-base-S), and (B) SPEDRE with cubic spline (SPEDRE) versus SPEDRE with smoothing spline (SPEDRE-S). Tests are performed on artificial networks of size 120-150 species. Each cell in the table shows the average results with standard deviation from independent tests with different network structures (n=3). Each dataset used in each test case was constructed by simulating the network using nominal reaction rates. Different levels of Gaussian noise were added to the simulated dataset: 5%, 10%, and 20%. The accuracy of each method was evaluated using species MRE, median PPE, maximum PPE, and weighted SSE.

Index	Reaction	Rate constant name	Value
1	GF + R → GF_R	k_uptake	14.39
2	inactNOX → NOX	kM_activNOX	0.1
3	inactNOX → NOX	kcat_activNOX	5.19
4	inactPI3K → PI3K	kM_activPI3K	0.09
5	inactPI3K → PI3K	kcat_activPI3K	6.81
6	NOX → inactNOX	k_deactNOX	3.19
7	PI3K → inactPI3K	k_deactPI3K	8.46
8	Environment → ROS	kM_NOX	0.15
9	Environment → ROS	kcat_NOX	13.56
10	ROS → Environment	kM_AntioxidantCapacity	1
11	ROS → Environment	kcat_AntioxidantCapacity	50
12	PTEN → PTENox	kM_ROS	0.09
13	PTEN → PTENox	kcat_ROS	0.72
14	PIP2 → PIP3	kM_PI3K	0.3
15	PIP2 → PIP3	kcat_PI3K	0.4
16	PIP3 → PIP2	kM_PTEN	0.3
17	PIP3 → PIP2	kcat_PTEN	0.5
18	Akt-cyto → Akt-mem	kcat_PIP3_Akt_cyto	0.4
19	Akt-mem → Akt-cyto	k_Akt_cyto	0.01
20	Akt-mem → Akt-p^{308}	kcat_PDK1_mem	0.6
21	Akt-p^{308} → Akt-cyto	kcat_PP2A_Akt_cyto	0.1
22	Akt-p^{308} → Akt-cyto	kM_PIP3_PDK1_cyto	0.5
23	PDK1-cyto → PDK1-mem	kcat_PIP3_PDK1_cyto	0.22
24	PDK1-mem → PDK1-cyto	k_PDK1_mem	0.12

Table S11 - Rate constant and nominal values in the Akt model. The prefix kM indicates a Michaelis-Mentent constant. The prefix kcat indicates a catalytic rate constant. As described in main text, the prefix "inact" denotes inactive species; the suffix "cyto" (or "mem") indicates cytosolic (or plasma membrane) localization; and the suffix "p^{308}" indicates phosphorylation at residue Thr^{308},which is crucial for Akt activation. The following species have initial concentration of 1.0: GF, R, NOX, PI3K, Environment, SOD, PTEN, PIP3, Akt-cyto, PP2A, PDK1. The remaining species have initial concentration of 0.0.

A

	Gaussian Noise	SPEDRE-base	SD	SRES	PSO	LM	GA
Run time	0%	0.11	13.16	69.42	272.70	272.70	15.97
	1%	0.09	13.55	66.88	278.09	278.09	17.45
	2%	0.08	15.34	62.69	270.17	270.17	10.55
	5%	0.09	15.53	68.72	272.19	272.19	15.47
	10%	0.09	14.39	57.81	328.20	328.20	8.95
	20%	0.08	11.31	67.53	282.88	282.88	21.83
Species MRE	0%	139.71	651.59	276.99	38.57	38.57	207.01
	1%	279.58	649.47	251.58	40.41	40.41	306.70
	2%	168.50	649.98	312.91	14.39	14.39	327.88
	5%	358.35	655.63	269.93	15.16	15.16	133.78
	10%	350.64	656.86	324.63	41.42	41.42	329.15
	20%	401.46	670.21	303.99	71.40	71.40	209.25
Median PPE	0%	37.95	5695.86	3046.31	86.67	86.67	74.49
	1%	90.47	5688.68	2679.22	75.60	75.60	94.92
	2%	69.06	5678.84	2711.93	68.13	68.13	99.54
	5%	96.48	5699.22	2082.84	98.97	98.97	74.86
	10%	181.24	5684.21	1423.99	94.33	94.33	95.58
	20%	86.93	5698.06	1913.27	91.70	91.70	97.77
Maximum PPE	0%	5119.05	394607.37	257379.30	39476.90	39476.90	30494.72
	1%	10393.03	453194.65	180024.46	31861.77	31861.77	7099.94
	2%	12395.93	486121.75	177815.97	13015.37	13015.37	17846.60
	5%	42036.94	344113.26	36698.60	17219.76	17219.76	1012.34
	10%	39882.51	443011.03	310906.61	49899.80	49899.80	5252.86
	20%	6139.83	388826.77	106555.93	12211.36	12211.36	12715.30
Weighted SSE	0%	2.42	81.14	5.06	0.04	0.04	5.21
	1%	5.55	71.54	2.85	0.05	0.05	2.13
	2%	4.17	71.84	3.17	0.07	0.07	6.16
	5%	18.14	85.09	5.42	0.31	0.31	6.81
	10%	33.44	79.79	5.48	1.23	1.23	5.26
	20%	19.45	88.39	8.29	4.74	4.74	11.87

B

	Gaussian Noise	SPEDRE	SRES_LM	PSO_LM	GA_LM	SPEDRE-base_SD	SRES_SD	PSO_SD	GA_SD
Run time	0%	5.11	6.31	3.53	16.53	9.03	0.42	0.17	4.72
	1%	4.14	5.80	0.91	16.95	0.61	0.44	0.38	0.59
	2%	3.00	5.91	1.98	2.72	0.61	0.22	0.17	3.97
	5%	5.20	9.63	2.42	6.20	0.64	0.28	0.17	6.30
	10%	6.73	16.08	22.19	14.36	0.63	0.22	0.23	7.34
	20%	19.09	15.56	16.27	18.88	0.53	0.44	0.20	1.13
Species MRE	0%	1.44E-04	1.37	39.19	0.41	130.16	276.55	38.57	206.99
	1%	3.51	6.74	40.34	6.96	233.96	251.38	40.41	307.27
	2%	7.00	7.14	14.28	6.99	168.48	312.89	14.39	327.85
	5%	17.90	17.89	15.34	17.43	354.81	269.86	15.17	104.31
	10%	36.01	34.74	40.35	33.38	348.85	324.63	41.42	249.89
	20%	71.72	66.45	71.28	117.40	394.03	303.83	71.40	209.25
Median PPE	0%	8.18E-04	1.46	86.61	0.52	34.08	3046.31	86.67	68.10
	1%	7.76	37.39	75.60	30.13	94.86	2679.22	75.60	94.47
	2%	21.61	16.64	68.09	17.42	69.05	2711.93	68.13	98.63
	5%	33.68	46.49	98.29	27.59	93.25	2082.84	98.97	30.65
	10%	60.94	98.97	98.94	88.71	182.22	1423.99	94.33	95.86
	20%	93.32	519.97	87.40	91.55	94.95	1913.29	91.70	97.77
Maximum PPE	0%	3.23E-02	9898.98	39281.20	86.64	5144.11	257379.30	39476.90	30494.72
	1%	352.71	5666.18	31862.44	326.44	10392.66	180024.46	31861.77	7099.94
	2%	587.63	9900.00	13003.46	584.17	12395.93	177815.97	13015.37	17273.97
	5%	9679.44	3308.92	17113.54	353.17	42036.82	36698.60	17219.76	198.35
	10%	9085.24	22857.83	49460.50	18064.79	39882.51	310906.61	49899.80	5275.28
	20%	8307.48	6621.28	12235.64	10563.50	6139.73	106555.62	12211.36	12715.30
Weighted SSE	0%	0.00	0.00	0.04	0.00	1.01	5.06	0.0391683	4.97244
	1%	0.01	0.02	0.05	0.02	3.94	2.85	0.0542735	2.09187
	2%	0.05	0.05	0.07	0.05	3.86	3.17	0.0706729	3.39356
	5%	0.29	0.30	0.31	0.29	16.87	5.42	0.30726	3.28937
	10%	1.18	1.18	1.23	1.19	32.58	5.48	1.23108	4.17792
	20%	4.75	4.83	4.74	6.32	15.73	8.28	4.74388	11.806

Table S12 - Comparison of standalone (**A**) and hybrid (**B**) rate parameter estimation methods applied to the Akt model (structure described in main text). Each dataset used in each test case was constructed by simulating the network using nominal reaction rates. Different levels of Gaussian noise were added to the simulated dataset: 0%, 1%, 2%, 5%, 10%, and 20%. The accuracy of each method was evaluated using species MRE, median PPE, maximum PPE, and weighted SSE.

REFERENCES

De Boor, C. (1994) *A Practical Guide to Splines*. Springer.

Hoops, S., *et al.* (2006) COPASI--a COmplex PAthway SImulator, *Bioinformatics*, **22**, 3067-3074.

Katare, S., Aditya Bhan, James M. Caruthers, W. Nicholas Delgass and Venkat Venkatasubramanian (2004) A hybrid genetic algorithm for efficient parameter estimation of large kinetic models, *Computers & Chemical Engineering*, **28**, 2569-2581

Moles, C.G., Mendes, P. and Banga, J.R. (2003) Parameter estimation in biochemical pathways: a comparison of global optimization methods, *Genome Res*, **13**, 2467-2474.

Rodriguez-Fernandez, M., Mendes, P. and Banga, J.R. (2006) A hybrid approach for efficient and robust parameter estimation in biochemical pathways, *Bio Systems*, **83**, 248-265.

Tewarson, R.P. (1980) On the use of splines for the numerical solution of nonlinear two-point boundary value problems, *BIT Numerical Mathematics*, **20**, 223-232.
